# Bracovirus Sneaks Into Apoptotic Bodies Transmitting Immunosuppressive Signaling Driven by Integration-Mediated eIF5A Hypusination

**DOI:** 10.3389/fimmu.2022.901593

**Published:** 2022-05-17

**Authors:** Gui-Fang Zhou, Chang-Xu Chen, Qiu-Chen Cai, Xiang Yan, Nan-Nan Peng, Xing-Cheng Li, Ji-Hui Cui, Yun-Feng Han, Qi Zhang, Jiang-Hui Meng, Hong-Mei Tang, Chen-hui Cai, Jin Long, Kai-Jun Luo

**Affiliations:** ^1^ School of Life Sciences, Yunnan University, Kunming, China; ^2^ Key Laboratory of the University in Yunnan Province for International Cooperation in Intercellular Communications and Regulations, Yunnan University, Kunming, China

**Keywords:** *Microplitis bicoloratus bracovirus*, Immunosuppression, eIF5A hypusination, viral integration, nucleocytoplasmic transport, apoptotic body, extracellular vesicles, viral re-integration

## Abstract

A typical characteristics of polydnavirus (PDV) infection is a persistent immunosuppression, governed by the viral integration and expression of virulence genes. Recently, activation of caspase-3 by *Microplitis bicoloratus bracovirus* (MbBV) to cleave Innexins, gap junction proteins, has been highlighted, further promoting apoptotic cell disassembly and apoptotic body (AB) formation. However, whether ABs play a role in immune suppression remains to be determined. Herein, we show that ABs transmitted immunosuppressive signaling, causing recipient cells to undergo apoptosis and dismigration. Furthermore, the insertion of viral–host integrated motif sites damaged the host genome, stimulating eIF5A nucleocytoplasmic transport and activating the eIF5A-hypusination translation pathway. This pathway specifically translates apoptosis-related host proteins, such as P53, CypA, CypD, and CypJ, to drive cellular apoptosis owing to broken dsDNA. Furthermore, translated viral proteins, such Vank86, 92, and 101, known to complex with transcription factor Dip3, positively regulated *DHYS* and *DOHH* transcription maintaining the activation of the eIF5A-hypusination. Mechanistically, MbBV-mediated extracellular vesicles contained inserted viral fragments that re-integrated into recipients, potentially *via* the homologous recombinant repair system. Meanwhile, this stimulation regulated activated caspase-3 levels *via* PI3K/AKT 308 and 473 dephosphorylation to promote apoptosis of granulocyte-like recipients Sf9 cell; maintaining PI3K/AKT 473 phosphorylation and 308 dephosphorylation inhibited caspase-3 activation leading to dismigration of plasmatocyte-like recipient High Five cells. Together, our results suggest that integration-mediated eIF5A hypusination drives extracellular vesicles for continuous immunosuppression.

**Graphical Abstract:**
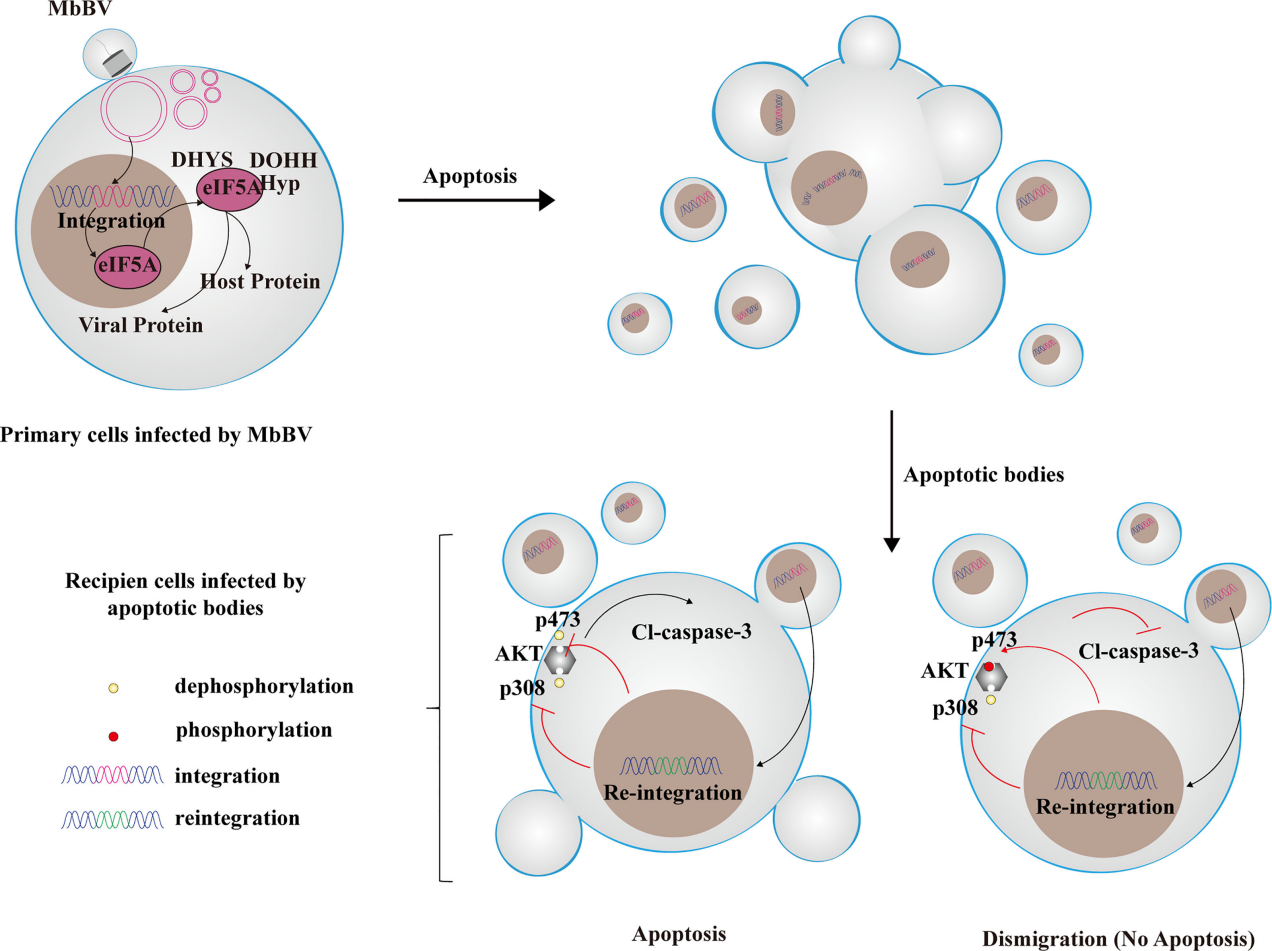


Bracovirus sneaks into apoptotic bodies where integration-mediated eIF5A hypusination drives immunosuppressive signaling transmission to recipients with different immunosuppressive characteristics. Reintegration of bracovirus fragments from apoptotic bodies affects different AKT phosphorylation sites, leading to different immune response from recipient cells.

## Highlights

- Recipient cells of MbBV-mediated apoptotic bodies showed dismigration and apoptosis
- MbBV integration activated the eIF5A-DHYS-DOHH hypusination pathway
- eIF5A hypusination drives immunosuppressive signaling transmission in apoptosis
- MbBV reintegration in recipient cells affected PI3K/AKT phosphorylation sites

## Introduction

Immunosuppression and related signaling transmission are important mechanistic elements of immune responses and need further investigation. In the virus–host immune system, the virus hijacks the host, hides in extracellular vesicles (EVs) to escape the immune response, spreads into new hosts, and generates progeny viruses (1). However, whether and how non-replicating viruses, like the *Polydnaviridae* (PDV) family, including the genera *Bracovirus* (BV) and *Ichnovirus* (IV), transmit immunosuppressive factors remain unknown. Herein, we attempt to explain how the non-replicating bracovirus persistently suppresses the immune system of lepidopteran hosts. *Microplitis bicoloratus bracovirus* (MbBV), a member of the PDV family, plays an essential role in suppressing immune response of lepidopteran hosts to protect the growth and development of parasitoid larvae in the host hemocoel (2, 3). Our recent studies have found that MbBV inhibition of the PI3K/AKT pathway induced hemichannel shutdown, triggering apoptosis and promoting the formation of apoptotic bodies (ABs) (4).

ABs (1–5 μm), the largest type of EVs, are vectors for intercellular communication, especially mediating immune responses (1, 5). Past studies have reported the use of these intercellular communication vector by viruses (6–9). Using EVs for virus transmission is an effective immune escape strategy, such as hepatitis C virus (HCV) (10). In addition, viruses can exploit EVs to regulate the immune system (11–13); the latent membrane protein-1 (LMP1) of human herpesvirus has been found in EVs and can effectively inhibit the immune response (14–16).

Viral integration into the host genome triggers an immune response (17, 18). The goal of viral integration is to transcribe viral genes and translate viral proteins. Notably, a damaged host genome also stimulates an immune response (19). Nevertheless, the most controversial observation is that viruses inhibit the host translation system while translating their own mRNAs. How viruses overcome this obstacle remains to be determined. Eukaryotic translation initiation factor 5A (eIF5A) plays an important role in modulating viral replication (20). Human immunodeficiency virus (HIV) was the first virus suggested to require eIF5A to regulate the expression of virion protein (Rev)-dependent nucleocytoplasmic transport (21–24). Ebola virus also requires eIF5A to regulate the secondary transcription of its genes by modulating the translation of the transcription factor VP30 (24, 25). Hypusine is essential for eIF5A activity, which is catalyzed by deoxyhypusine synthase (DHYS) (26, 27) and deoxyhypusine hydroxylase (DOHH) (28–30). However, little is known about the interaction between viral integration and eIF5A hypusination.

In this study, we address two major gaps in understanding the mechanism underlaying immunosuppression: (1) how the integration of bracovirus drives apoptosis, and (2) how ABs transmit immunosuppressive signaling. We demonstrated that bracovirus sneaks into ABs where integration-mediated eIF5A hypusination drives immunosuppressive signaling transmission to recipient, with different immunosuppressive characteristics, such as apoptosis and dismigration. Reintegration of the bracovirus fragments of ABs alters AKT phosphorylation sites and immune response in recipient cells.

## Results

### Bracovirus-Induced ABs Transmit Immunosuppressive Signaling

**Figure 1:**
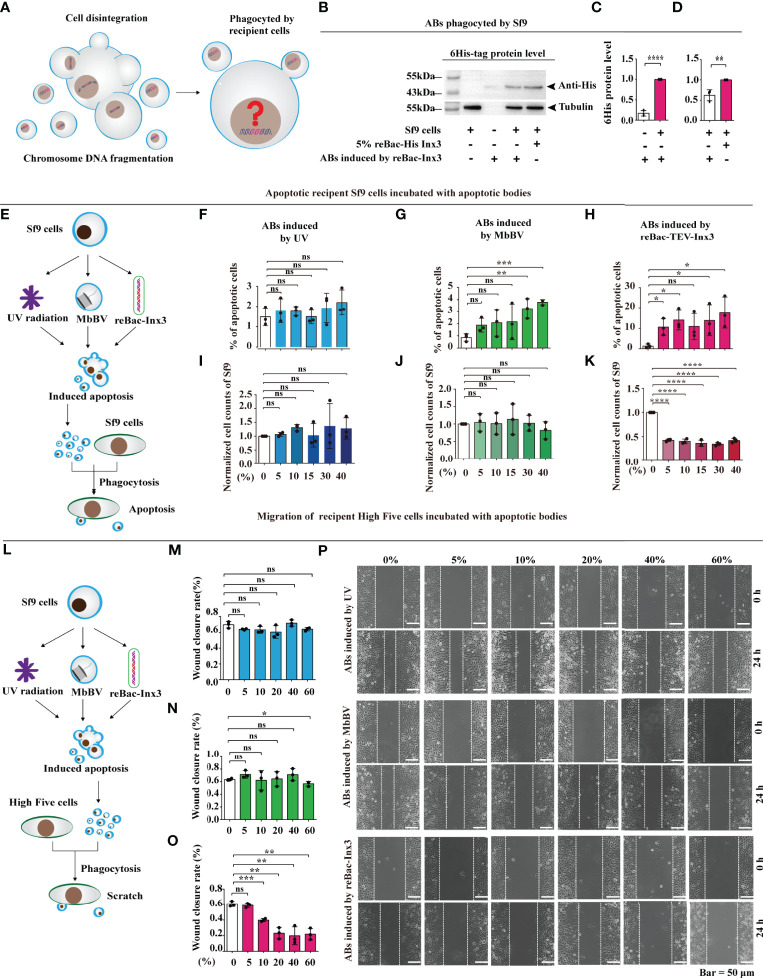
Bracovirus-induced ABs transmit immunosuppressive signaling. **(A)** Schematic illustration of the fate of the extracellular vesicles phagocytosed by the recipient cells. **(B–D)** Western blot detection of 6 × His labeled AB of the recipient cells. **p < 0.01, ****p < 0.0001, error bars represent SEM. Unpaired Student’s *t*-test with Holm-Sidak method for multiple *t* test; n = 3. **(E–K)** Formation of ABs induced by MbBV mediated apoptosis of recipient Sf9 cells. Flowchart showing the formation of ABs generated by UV, MbBV, reBAc-His-Inx3 **(E)**. The detection of apoptotic recipient Sf9 cells incubated with UV-induced AB **(F)**, MbBV-induced AB **(G)** and re-Bac-Inx3-induced AB compared with different concentration **(H)**. The detection of cell numbers of recipient Sf9 cells after incubation with UV-induced AB **(I)**, MbBV-induced AB **(J)** and re-Bac-Inx3-induced AB compared with different concentration **(K)**. *p < 0.05; **p < 0.01; ***p < 0.001; ****p < 0.0001; ns, no significant difference; error bars represent SEM. Unpaired Student’s *t*-test with Holm-Sidak method for multiple *t* test; n = 3. **(L–P)** Formation of ABs mediated by MbBV inhibited the migration of recipient High Five cells. The flowchart of formation of ABs generated by UV, MbBV reBAc-His-Inx3 **(L)**. The detection of migration recipient High Five cells incubated with UV-induced AB **(M)**, MbBV-induced AB **(N)**, and reBac-Inx3-induced AB **(O)**. Scratch assays in recipient High Five cells **(P)**. Scale bar, 50 μm. *p < 0.05, **p < 0.01, ***p < 0.001, ns, no significant difference, error bars represent SEM. Unpaired Student’s *t*-test with Holm-Sidak method for multiple *t* test; n = 3. **See also** **Figure S1**.

We have previously shown that MbBV induces AB formation by shutting down hemocyte hemichannels to activate caspase-3 and cleave Innexins (Inxs), triggering apoptotic cell disassembly (4). However, the biological role of MbBV-induced ABs in immunosuppression remains poorly understood (**Figure 1A**). To determine whether MbBV suppresses the immune response *via* AB phagocytosis, we used ABs-induced by reBac-Inx3 (4, 31, 32) as a positive control, detectable using 6 × His tag (**Figure S1A**) to allow us to determine whether ABs can be phagocytosed by Sf9 cells. The ABs induced by reBac-Inx3 were divided equally (**Figure S1B**) for direct protein quantification from the ABs, while the other was incubated with Sf9 cells (**Figures 1B–D**). The 6 × His tag expression was significantly higher in Sf9 cells incubated with equal ABs (**Figure 1C**), but lower in ABs-incubated Sf9 cells than in reBac-Inx3-infected Sf9 cells (**Figure 1D**), suggesting that viruses from ABs were amplified in recipient Sf9 cells. Thus, ABs induced by reBac-Inx3 can be used as a positive control of ABs mediated by MbBV.

Accordingly, we treated Sf9 cells using, UV radiation, MbBV, and reBac-Inx3 and collected ABs using gradient centrifugation (**Figure S1C**), to confirm whether ABs promote AB formation (**Figures 1E** and **S1D**). Expectedly, the total number of apoptotic cells incubated with different concentrations of UV-induced ABs was not significant (**Figure 1F**). The MbBV-induced ABs promoted Sf9 cell apoptosis when the concentration exceeded 30% (**Figure 1G**); similarly, apoptosis of Sf9 cells was significantly increased by reBac-Inx3-induced ABs (**Figure 1H**). We also detected the effect of different ABs on cell number and found no significant differences in UV- and MbBV-induced ABs (**Figures 1I, J**). In contrast, a significant decrease in the cell number was observed from 5–40% reBac-Inx3-induced ABs (**Figure 1K**). These results show that the MbBV-induced ABs can promote the apoptosis of recipient Sf9 cells.

Since the disturbance of dynamic cytoskeleton regulation is one of the crucial phenotypes of bracovirus infection (33–36), ABs induced by UV, MbBV, and reBac-Inx3 were incubated with scratch High Five cells in a concentration gradient manner for 24 h (**Figure 1L**). UV-induced ABs did not affect the wound healing rate of High Five cells with the increase in concentration (**Figures 1M, P**). In contrast, ABs induced by MbBV and reBac-Inx3 inhibited the migration of High Five cells at higher concentrations, MbBV 60% (**Figure 1N**) and reBac-Inx3 from 10–60% (**Figure 1O**). Results confirmed that MbBV-induced ABs could inhibit the dynamic cytoskeleton regulation in High Five cells. Our data suggest that MbBV-induced ABs transmit immunosuppressive signaling, thereby inducing recipient cell apoptosis and dismigration.

We investigated how MbBV regulates primarily infected cells and how are viral proteins are translated to determine these functions of ABs.

### Bracovirus Primarily Integrates Host *Spodoptera litura* Genome *via* Host Integrated Motif

Some bracoviral genomes showed that they can be integrated into the host genome *via* host integrated motif (HIM), as in the cases of *Microplitis demolitor bracovirus* (MdBV) (37) and *Cotesia congregata bracovirus* (CcBV) (38). To determine the viral integrated sequence in host genome, we sequenced the natural parasitized *S. litura* hemocytes and MbBV-infected Spli221 cells, to obtain the bracovirus-integrated *S. litura* genome and compared genome of MbBV with that of MdBV and CcBV to identify the HIM of MbBV (**Tables S1, S2**). Viral HIM was compared with bracovirus-integrated *S. litura* DNA sequence to isolate viral DNA-HIM-host DNA containing the hybrid fragments. Finally, by mapping fragments to 31 *S. litura* chromosomes (39), the position of integrated MbBV DNA was identified and quantified (**Figure S2A**).

**Figure 2:**
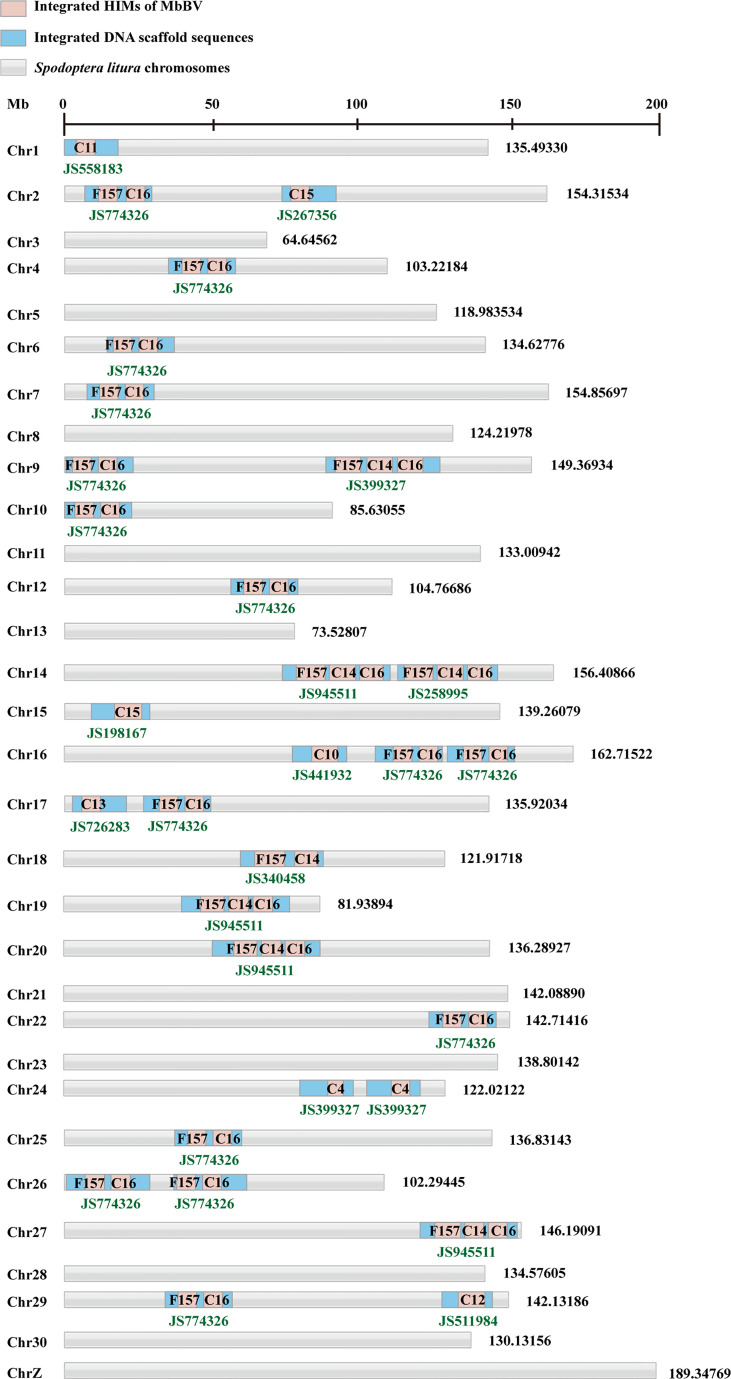
Bracovirus primarily integrates host *Spodoptera litura* genome *via* host integrated motif. **See also** **Figure S2**, **Tables S1** and **S2**.

The structure of MbBV HIMs was predicted, and sequences containing the HIM junction were selected. All 17 HIMs had similar structures, including two homologous boundary sites (GAAAATTTC) on both the 5′ and 3′ terminals, one homologous junction 1 (CTAGT), one homologous junction 2 (ACTAGG), and a non-homologous loop (**Figure S2B**). A total of 12 hybrid fragments, 6 containing MbBV DNA, HIM-Junction 1, and host chromosome, and 6 containing host DNA, HIM-Junction 2, and MbBV DNA (**Figure S2C**), were mapped to the genome of *S. litura*. Viral integrated DNA into host cells were identified (**Figure 2**). Overall, viral DNA did not integrate into all host chromosomes; integrated MbBV DNA was found only in 21 out of 31 chromosomes, while the remaining 10 chromosomes, namely Chr 3, 5, 8, 11, 13, 21, 23, 28, 30, and Z, showed no viral integration. Compared with the MbBV genome (40), 8 out of 17 circles and a fragment were integrated, namely C4, C10, C11, C12, C13, C14, C15, C16, and F157 (**Figure 2**).

Primers were designed to amplify HIM-C11 in Chr 1, HIM-C14 in Chr 9, HIM-C10 in Chr16, and HIM-C16 in Chr 19, in both parasite hemocytes and MbBV-infected Spli221 cells. The viral integrated genes *vank86*, *99*, *100*, *101*, *PTP 102*, and *vank76*, were detected in the host hemocytes parasitism, not non-parasitism (**Figure S2D**) to further confirm the above results. In conclusion, the partial MbBV is integrated into genome of host *via* 17 HIM in 21 host chromosomes; at least 8 of 17 MbBV circles and a fragment contained HIM sites and integrated into the host chromosomes.

### Bracovirus Integration Triggers eIF5A Hypusination

**Figure 3:**
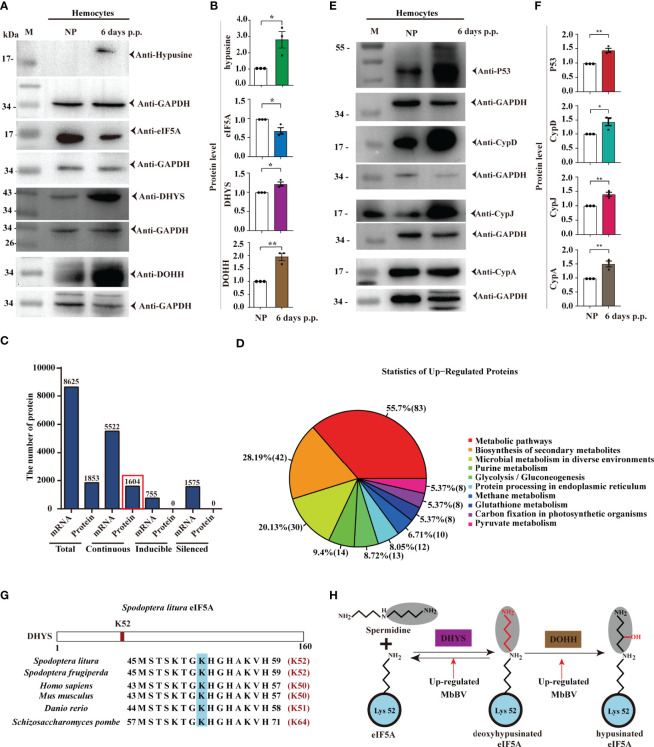
Bracovirus integration triggered eIF5A hypusination. **(A, B)** Bracovirus integration increases the expression of hypusination, DHYS, and DOHH. *p < 0.05, **p < 0.01, error bars represent SEM. Unpaired Student’s *t*-test with Holm-Sidak method for multiple *t* test; n = 3. **(C)** Expression of proteins and mRNAs under MbBV integration hypusination. Total, continuous, inducible, and silenced mRNA and proteins are identified. **(D)** Upregulated proteins are enriched in 10 pathways. **(E, F)** Upregulated proteins, P53, CypD, CypJ, and CypA, related to apoptosis, are detected. *p < 0.05, **p < 0.01, error bars represent SEM. Unpaired Student’s *t*-test with Holm-Sidak method for multiple *t* test; n = 3. **(G)** eIF5A hypusination sites. **(H)** MbBV increased DHYS and DOHH expression and modified eIF5A hypusination. **See also** **Figure S3** and **Table S3**.

Previously, we reported that MbBV inhibits the eIF4E-eIF4A axis (3) *via* viral Vank proteins interacting with host Dip3 (41). We speculated that there exists another translation pathway that may translate viral proteins. eIF5A is required for HIV and Ebola viral replication (23–25). Hemocytes were collected to detect the eIF5A-DHYS-DOHH pathway, showing that eIF5A was hypusinated; DHYS and DOHH expression were upregulated 6 days post parasitism (**Figures 3A, B**).

Next, we performed transcriptomic and proteomic analyses to identify the translated proteins of *S. litua* hemocytes triggered by eIF5A hypusination under natural parasitization. iTRAQ was performed using hemocytes, and MS/MS data were analyzed with transcription mRNA data (42) together, from two proteomes, M and S (M: Parasitized hemocytes; S: Non-parasitized hemocytes) and three transcriptomes, All, M, and S (All: Mixture, M and S as above) (**Figures S3A-C**). A total of 1853 proteins and 8625 mRNAs were separated into three classifications: continuous expression 5522 mRNAs (found in the three pools, All, M, and S) and 1604 proteins (found in two pools M and S); inducible 755 mRNAs (in All and M) and silenced 1575 mRNAs (in All and S) (**Figure 3C**).

Based on previous data showing that ribosomes are stalled in the absence of hypusinated-eIF5A when proline (P) and glycine (G), or charged amino acids [aspartic acid (D^-^), glutamic acid (E^-^), lysine (K^+^), arginine (R^+^), and histidine (H^+^)] are located upstream of the P-aminocarbonyl tRNA (43), we further identified the involved proteins. Our analysis revealed that all upregulated proteins were involved in 10 pathways (**Figure 3D**). Over 35% of 7 amino acids (P, G, D, E, K, R) were separated (**Figure S3D**), while 10 proteins, namely P53, CypA, CypD, CypJ, Vank86, Vank92, Vank101, eIF5A, DHYS and DOHH, were highly expressed and used as hallmarks of hypusination (**Table S3**). Accordingly, we identified that the cellular apoptosis-related proteins, P53, CypD, CypJ, and CypA, were highly expressed under hypusination 6 days post parasitization (**Figures 3E, F**).

Conserved K52 was modified by hypusination in *S. litura*, and its vicinity was highly conserved from yeast to humans (44, 45) (**Figure 3G**). eIF5A is hypusinated through a two-enzyme cascade, in which an aminobutyl group from the polyamine spermidine is first covalently attached to lysine 52 of eIF5A by DHYS and then DOHH (46). Moreover, MbBV upregulated DHYS and DOHH expression (**Figure 3H**). These results demonstrated that natural parasitism of *M. bicoloratus* promoted hemocyte hypusination of eIF5A and triggered hypusination-dependent protein translation by blocking eIF4E-eIF4A (3, 47) translation during immunosuppression of *S. litura*.

### Viral-Hypusination-Dependent Proteins Positively Regulated *DHYS* and *DOHH*

**Figure 4:**
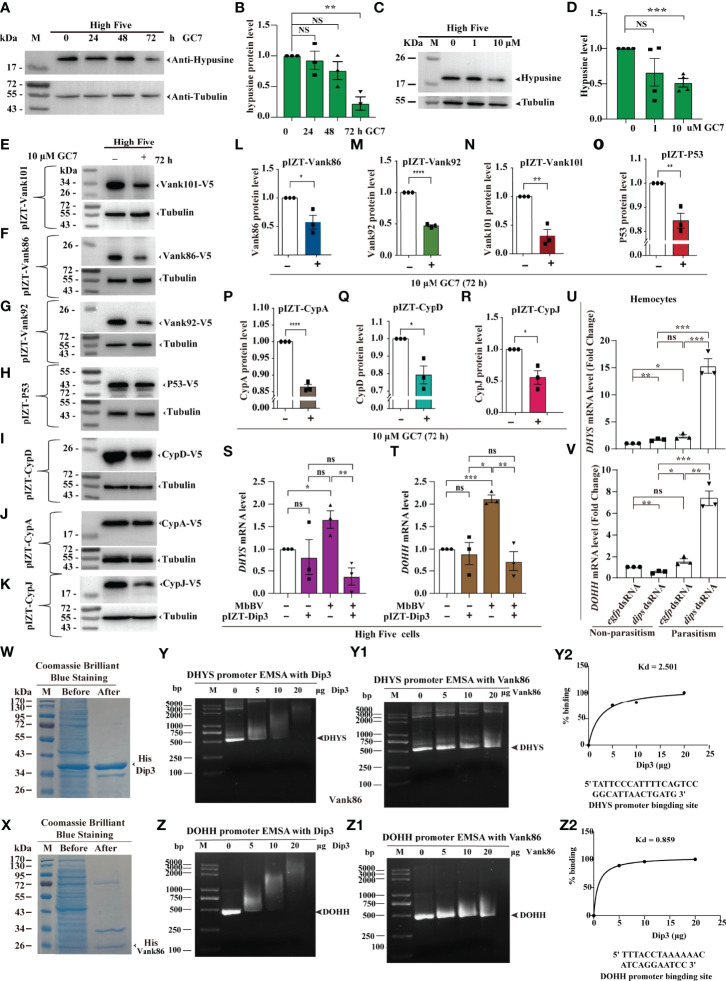
Viral-hypusination-dependent proteins positively regulated *DHYS* and *DOHH.* **(A-R)** GC7 inhibited hypusination-dependent protein expression. GC7 inhibition of hypusination at different times **(A, B)** and different dosages **(C, D)**; GC7 inhibited the ectopic expression of the integrated proteins, Vank86, 92, 101, P53, CypA, CypD, and CypJ **(E-K)**; Column summarizes the level of Vank 86, 92, 101, P53, CypA, CypD, and CypJ in High Five cells, normalized to the total protein level **(L-R)**. *p < 0.05; **p < 0.01, ***p < 0.001, ****p < 0.0001, ns, no significant difference, error bars represent SEM. Unpaired Student’s *t*-test with Holm-Sidak method for multiple *t* test; n = 3. **(S-V)** Viral-hypusination-dependent proteins positively regulated *DHYS* and *DOHH* transcription. Ectopic expression of Dip3 decreased the expression of *DHYS* and *DOHH* infected with MbBV **(S, T)**; *in vivo*, parasitism plus *dip3* dsRNA increased the expression of *DHYS* and *DOHH* **(U, V)**. *p < 0.05, **p < 0.01, ***p < 0.001, ****p < 0.0001, ns, no significant difference, error bars represent SEM. Unpaired Student’s *t*-test with Holm-Sidak method for multiple *t* test; n = 3. (**W-Z2**) Electrophoretic mobility shift assay (EMSA) to detect the promoter of *DHYS* and *DOHH* interaction with Dip3. Purified Dip3 and Vank86 proteins **(W, X)**; DHYS **(Y)** or DOHH **(Z)** promoter EMSA with Dip3, Vank86 as a control protein (**Y, Z1**); DHYS (**Y2**) and DOHH (**Z2**) presented high affinity. **See also** **Figure S4**.

MbBV-integrated genes were highly expressed following hypusination. To determine whether overexpression proteins are dependent on eIF5A hypusination, we used DHYS inhibitor, N1-guanyl-1,7-diaminoheptane (GC7), to block the eIF5A hypusine signaling pathway. Experimental results showed that GC7 inhibited the hypusine modification of eIF5A at a concentration of 10 uM during a 72-h treatment (**Figures 4A–D**). Inhibition of ectopic expression of the V5-fusion proteins in the High Five cells using 10 µM GC7 for 72 h, Vank proteins, Vank86, 92, 101, were inhibited by GC7, presenting the same pattern as hypusine-dependent proteins, namely P53, CypD, CypA, and CypJ (**Figures 4E–R**). The data above demonstrate that MbBV regulates the translation of seven host and viral proteins rich in proline, glycine, and amino acids through hypusine (**Table S3**).

Previous research has shown that Vank-Dip3 inhibits the transcription of eIF4E and its regulated genes (41); however, whether and how Dip3 regulates DHYS and DOHH during Vank protein expression remains nebulous. Accordingly, we performed RT-qPCR to determine how Dip3 regulates the transcription of *DHYS* and *DOHH*. MbBV promoted the transcription of *DHYS* and *DOHH*, which was not predicted to be inhibited; then overexpression Dip3 decreased the transcription of *DHYS* and *DOHH* triggered by MbBV (**Figures 4S, T**). Next, to determine whether Dip3 indeed inhibited the transcription of *DHYS* and *DOHH*, we designed parasitism plus RNAi to block Dip3, which increased *DHYS* and *DOHH* transcription compared with control *egfp* dsRNA and *dip3* dsRNA only non-parasitism (**Figures 4U, V**). The results showed that MbBV positively regulated *DHYS* and *DOHH* transcription, thus we hypothesized that Dip3 may be a negative regulator of *DHYS* and *DOHH*.

Based on previous observations, we hypothesized that Dip3 interacted with the promoter DNA of *DHYS* and *DOHH* to inhibit transcription. Furthermore, an electrophoretic mobility shift assay (EMSA) helped us demonstrate. Dip3 and Vank86 proteins were expressed and purified (**Figures 4W, X**); promoter fragments of *DHYS* and *DOHH* were predicted and cloned (**Figures S4A, B**). EMSA showed that Dip3 was bound to the promoter of both genes, not Vank86 (**Figures 4Y, Z**2). The data supported our hypothesis that Dip3 negatively regulated *DHYS* and *DOHH via* binding to their promotors. In conclusion, these results showed that viral-hypusination-dependent protein positively regulated *DHYS* and *DOHH* transcription.

### Bracovirus Activates Hypusination *via* eIF5A Nuclear-Cytoplasmic Transport

**Figure 5:**
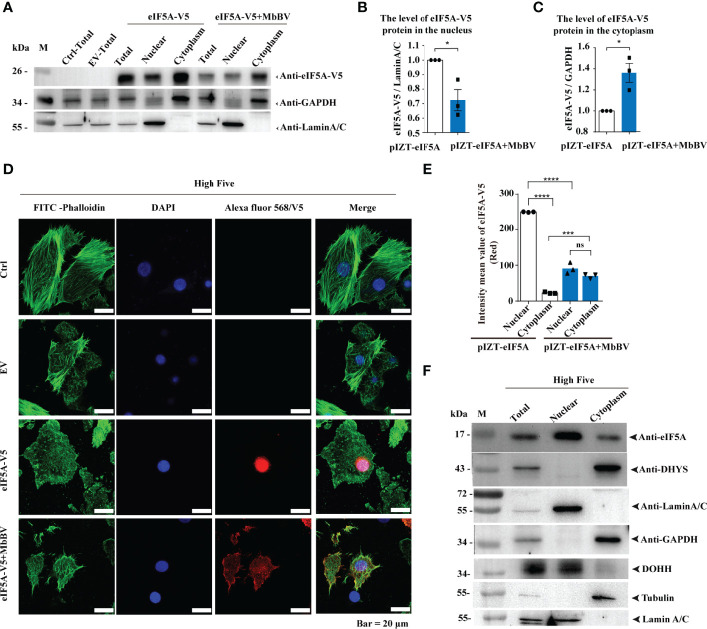
Bracovirus activated hypusination *via* eIF5A nuclear-cytoplasmic transport. **(A–C)** Western blotting was used to detect the effect of MbBV on the localization of ectopically expressed eIF5A in High Five cells. The column summarizes the level of eIF5A, normalized to nucleoprotein and cytoplasmic protein respectively. *p < 0.05, error bars represent SEM. Unpaired Student’s *t*-test with Holm-Sidak method for multiple *t* test; n = 3. **(D, E)** Immunofluorescence was used to detect the effect of MbBV infection on the localization of V5-eIF5A in High Five cells. Scale bar, 20 μm. The column summarizes the mean fluorescence intensity. ***p < 0.001, ****p < 0.0001, ns, no significant difference, error bars represent SEM. Unpaired Student’s *t*-test with Holm-Sidak method for multiple *t* test; n = 3. **(F)** Nuclear protein isolated to identify the localization of eIF5A, DHYS and DOHH. **See also** **Figure S5**.

Next, we investigated how MbBV regulates hypusine modification. Hypusine modification of eIF5A dictates its location in the cytoplasm, where it is required for protein synthesis (48). Ectopic expression of eIF5A, DHYS, and DOHH did not change eIF5A hypusination, and hallmark proteins, P53, CypA, and CypD, were expressed following the same pattern, except for CypJ, which overexpressed DOHH (**Figures S5A, B**), suggesting that these protein level did not affect eIF5A hypusination. We next determined whether MbBV modulated its protein synthesis function by altering hypusine localization. We performed western blotting to detect the localization of hypusine modification-related factors of eIF5A in High Five cells under MbBV stimulation. eIF5A was primarily located in the nucleus and transported to the cytoplasm during MbBV infection (**Figures 5A–C**). Furthermore, immunofluorescence analysis showed that eIF5A was predominantly localized in the nucleus, while during MbBV infection, eIF5A is transported to the cytoplasm (**Figures 5D, E**). DHYS was only located in the cytoplasm, while DOHH was only located in the nucleus of the High Five cells (**Figure 5F**). Immunofluorescence analysis showed that MbBV did not change the location of DHYS and DOHH (**Figure S5C**). These results collectively demonstrated that MbBV acts by facilitating eIF5A transport from the nucleus to the cytoplasm.

### Bracovirus Integration-Mediated Hypusination Drives Persistence Apoptosis

Hypusination is required for specific protein translation and is accompanied by cell apoptosis, as evidenced by the expression of apoptosis-related proteins, P53, CypA, CypD, and CypJ. The next question is whether hypusination is required for maintaining persistent apoptosis. We utilized parasitization to activate bracovirus integration-mediated hypusination (BIMH) and an RNAi-mediated silencing approach to inhibit eIF5A hypusination and detect apoptotic cells.

**Figure 6:**
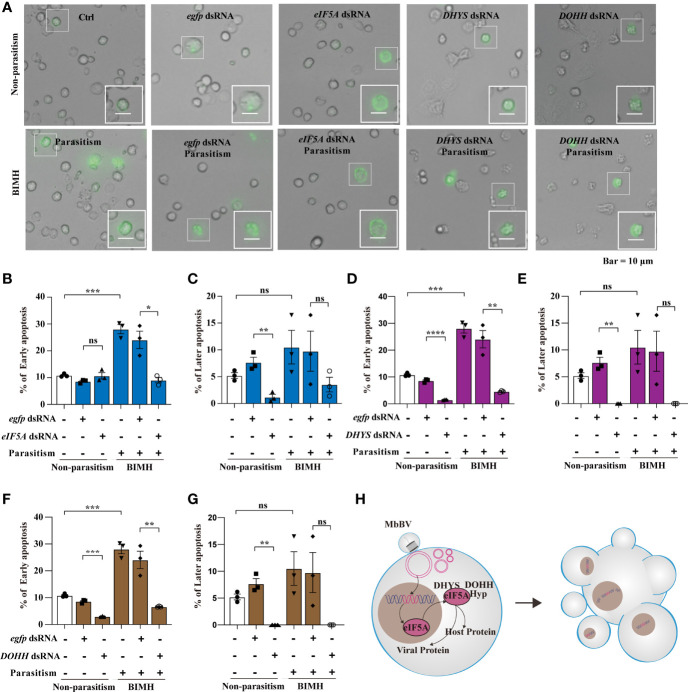
Bracovirus integration-mediated-eIF5A-hypusination drives persistence apoptosis. **(A)** The effect of RNAi to inhibit hypusine on apoptosis was studied by detecting the apoptotic hemocytes with annexin V-conjugated FITC and PI. Inverted fluorescence microscopy illustrates the early apoptosis labeled with AnnexinV–FITC (shown in green). Scale bar, 10 μm. This was observed in parasitism under hypusination condition. **(B–G)** The early and late apoptosis of *eIF5A* dsRNA, *DHYS* dsRNA and *DOHH* dsRNA plus parasitization. *p < 0.05, **p < 0.01, ***p < 0.001, ****p < 0.0001, ns, no significant, error bars represent SEM. Unpaired Student’s *t*-test with Holm-Sidak method for multiple *t* test; n = 3. **(H)** Illustration of the progress of hypusination triggered by integration of genome promoter in persistent apoptosis. **See also** **Figure S6**.

*In vivo*, inhibiting eIF5A decreased early apoptosis only in the BIMH condition (**Figures 6A, B**), while inhibition of *DHYS* and *DOHH* decreased early apoptosis in both non-parasitism and BIMH (**Figures 6D, F**); however, in later apoptosis, there were significant differences in non-parasitism and no differences were observed in the BIMH (**Figures 6C, E, G**), suggesting that hypusination inhibition halted early apoptosis, a mechanism required to maintain persistent apoptosis.

We also examined the effect of the hypusination pathway on development. The head capsule development experiment showed that its development was significantly inhibited by *eIF5A* dsRNA at day 3, 8 and 9 after feeding (**Figure S6A**). However, there was no significant difference between treatment groups with eIF5A RNAi after parasitism and the *egfp* dsRNA control group (**Figure S6B**). The development was significantly inhibited by *DHYS* dsRNA at 9 days after feeding, while no significant difference was observed between groups with silenced *DHYS* after parasitism (**Figures S6C, D**). Furthermore, in *DOHH*-silenced conditions, there were no significant differences, except for day 9 after parasitism where the development was significantly increased (**Figures S6E, F**). These data indicated that the silencing of the hypusine pathway affected the immunosuppression mediated by *M. bicoloratus* natural parasitism and caused no significant difference in the head capsule width in *S. litura*.

These results collectively showed that integrated MbBV-mediated eIF5A hypusination is required for driving cell (**Figure 6H**).

### Bracovirus Fragments in ABs Re-Integrate Into Recipient Cells

MbBV-mediated innexin-hemichannel closure causes apoptotic cell disassembly; thereby facilitating MbBV-induced apoptosis *via* eIF5A hypusination. However, reasons behind recipient cells presenting apoptosis and dismigration during immunosuppression, and whether bracovirus-mediated ABs can transmit viral genes to recipient cells remain to be determined.

**Figure 7:**
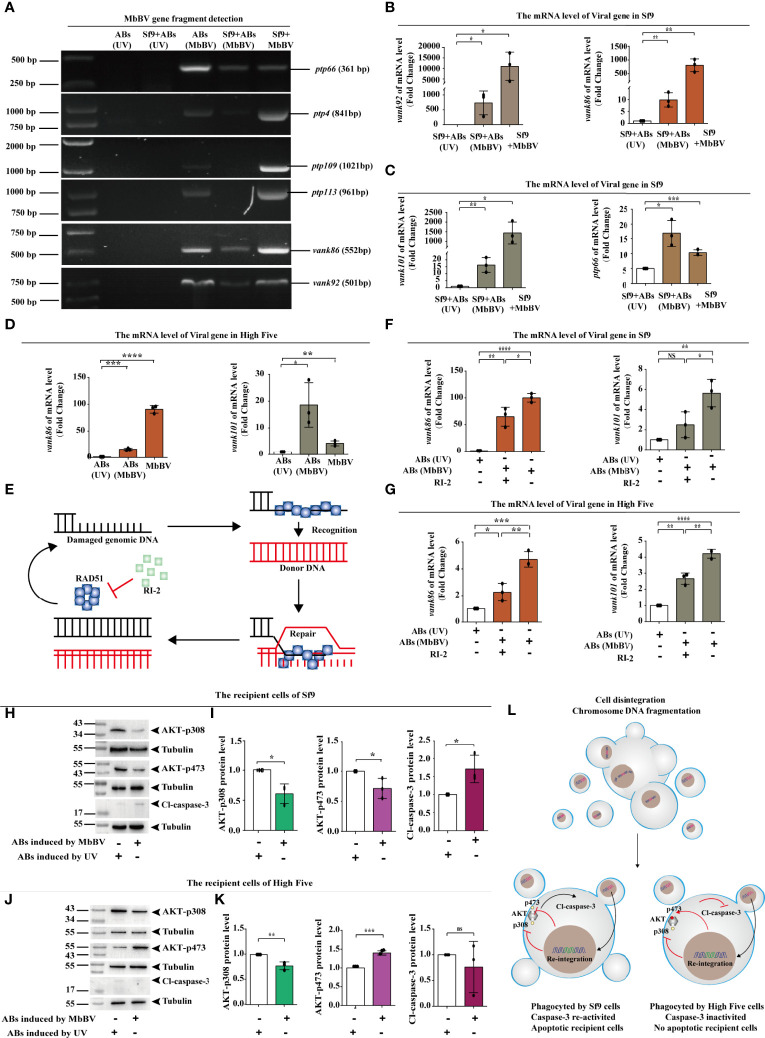
Bracovirus fragments in extracellular vesicles re-integrate into recipient cells. **(A)** Detection of viral gene fragments in ABs and Sf9 recipient cells. **(B**, **C)** Detection of transcription of viral genes, *vank92*, *vank86, vank101, and PTP66* in Sf9 recipient cells. *p < 0.05, **p < 0.01, ***p < 0.001, error bars represent SEM. Unpaired Student’s *t*-test with Holm-Sidak method for multiple *t* test; n = 3. **(D)** Detection of transcription of viral genes, *vank86 and vank101* in High Five recipient cells. *p < 0.05, **p < 0.01, ***p < 0.001, ****p < 0.0001, error bars represent SEM. Unpaired Student’s *t*-test with Holm-Sidak method for multiple *t* test; n = 3. **(E)** Homologous recombination repair system mediated by RAD51. **(F, G)** Detection of viral genes transcription in recipient cells after inhibition of RAD51. *vank86* and *vank101* in recipient Sf9 cells **(F)** and in the recipient High Five cells **(G)**. *p < 0.05, **p < 0.01, ***p < 0.001, ****p < 0.0001, ns, no significant difference, error bars represent SEM. Unpaired Student’s *t*-test with Holm-Sidak method for multiple *t* test; n = 3. **(H, I)** Sf9 recipient cells showed decreased expression of AKT-p308 and AKT-p473 **(H)**, increase Cl-caspase-3 **(I)** using western blotting. *p < 0.05, ns, no significant, error bars represent SEM. Unpaired Student’s *t*-test with Holm-Sidak method for multiple *t* test; n = 3. **(J, K)** High Five recipient cells showed decreased expression of AKT-p308 and increased expression of AKT-p473 **(J)** as well as inhibited Cl-caspase-3 **(K)**. **p < 0.01, ***p < 0.001, ns, no significant, error bars represent SEM. Unpaired Student’s *t*-test with Holm-Sidak method for multiple *t* test; n = 3. **(L)** Mechanism of re-integrated involvement in apoptotic recipient Sf9 cells and inhibition of migration in the recipient High Five cells.

We speculated that viral gene fragments are transmitted between cells by ABs as carriers. We used PCR to detect viral gene fragments in the genome of Sf9 recipient cells incubated with MbBV-induced ABs and found that *vank86*, *vank92*, and *ptp66* were present in recipient cell genome (**Figure 7A**). The existence of viral genes in the recipient cell genome indicates these genes may be transcribed. Therefore, we detected the transcription of viral genes, including *vank86*, *vank92*, *vank101*, and *ptp66*, in Sf9 recipient cells (**Figures 7B, C**). The transcription of viral genes, including *vank86* and *vank101*, was detected in High Five recipient cells (**Figure 7D**). However, MbBV mediates dsDNA breaks (49) that can damage the HIM; thus, how viral fragments without HIM integrate into the genome of recipient cells is currently unknown. The homologous recombination repair system mediates the integration of exogenous DNA (50), especially when these exogenous DNA fragments carry homologous DNA sequences that can improve the integration efficiency (**Figure 7E**) (51). Thus, we equally divided ABs into two parts. Half of the ABs were treated with RI-2 (inhibitor of RAD51), while the other half was not. Recipient cells incubated with UV-induced ABs were used as a negative control.

After incubating the ABs with recipient cells for 72 h, the transcription levels of the viral genes, *vank86* and *vank101*, were significantly decreased following RI-2 treatment in both Sf9 and High Five recipient cells; compared with the negative control, the transcription level of the RI-2 treatment group was significantly increased (**Figures 7F, G**). These results suggested that AB-mediated viral gene transfer was related to the homologous recombination repair system but was not completely dependent on the homologous recombination repair system.

Next, we investigated the biological role of viral fragment reintegration. We have previously found that blocking the PI3K/AKT signaling pathway is essential for MbBV gene products to perform immunosuppressive function (4). To demonstrate that the virus genes are responsible for the physiological and biochemical changes in recipient cells, we examined the activation of the PI3K/AKT signaling pathway in both Sf9 and High Five recipient cells. The phosphorylation levels of AKT- p308 and AKT-p473 were significantly reduced in Sf9 recipient cells, and Cl-caspase-3 was significantly activated (**Figures 7H, I**). Similarly, AKT phosphorylation was also detected in the High Five recipient cells and found that only AKT-p308 was dephosphorylated. In contrast, AKT-p473 presented high phosphorylation, and Cl-caspase-3 did not significantly differ in High Five recipient cells (**Figure 7K**). Considering that AKT requires AKT-p308 and AKT-p473 co-phosphorylation to perform signal transduction, only AKT-p308 dephosphorylation affected transduction, as evidenced by the inhibition of High Five cell migration.

In summary, viral gene products were transcribed and expressed after viral gene fragments re-integrate into recipient cells through ABs. Activated caspase-3 promoted Sf9 recipient cell apoptosis, and pAKT-308 dephosphorylation promoted High Five recipient cell dismigration. These observations indicate that gene products could still exert immunosuppression by regulating the pI3K/AKT signaling pathway (**Figure 7L**).

## Discussion

Immunosuppressive signaling transduction is a novel aspect underlying immunity mechanisms. The PDV virus family usually cannot replicate after infecting host cells due to their special replication and symbiosis mechanism (52–56). Although PDV can cause persistent immunosuppression in lepidopterans, the specific mechanism remains unclear. This study explored the effect of MbBV on immunosuppressive signaling transmission. The results showed that MbBV-mediated ABs transmitted viral fragments to recipient cells, causing persistent immunosuppression. MbBV integration triggered eIF5A hypusination to translate apoptosis-related and viral proteins; the former maintained persistent apoptosis, the latter positively activated eIF5A hypusination pathway. MbBV induced dsDNA breaks in viral fragments delivered to recipient cell *via* ABs, which reintegrated to regulate PI3K/AKT phosphorylation to suppress immune responses, leading to recipient cell apoptosis and dismigration. To the best of our knowledge, this report is the first to explore the mechanism underlying immunosuppression transmission by bracovirus-mediated ABs.

Bracovirus induce apoptosis in granulocytes and loss of adhesion in plasmatocyte (49, 57), and further disassembly of apoptotic cells (4). In our research, Sf9 cells, which have low–level of activated caspase-3, were characterized to be like of granulocytes, while High Five cells, which have inactive caspase-3, were characterized to be more like plasmatocyte (58). Using these two cell types, we provide evidence that MbBV-infected Sf9 cell-derived ABs can inhibit cell migration after phagocytosis by High Five recipient cells. Similarly, ABs can induce apoptosis after being engulfed by Sf9 recipient cells. The key to the normal operation of the immune function lies in the activation of immune-related signaling pathways (59). However, our previous studies have shown that MbBV induced immunosuppression by inhibiting the PI3K/AKT signaling pathway (4). The phosphorylation sites p308 and p473 of PI3K/AKT in Sf9 recipient cells, co-incubated with ABs from infected Sf9 cells, were inhibited, thereby promoting Cl-caspase-3 activation. Meanwhile, the p308 phosphorylation site of PI3K/AKT in the High Five recipient cells was inhibited. Activation of the PI3K/AKT signaling pathway requires co-phosphorylation of two phosphorylation sites (60). Therefore, the inhibition of either two or a single phosphorylation site can lead to immune inactivation. This may explain the apoptosis in granulocytes-like Sf9 cells and loss of adhesion in plasmacytes-like High Five cells causing dismigration after the cells engulfed MbBV-mediated ABs.

MbBV integration has been reported to trigger eIF5A hypusination. In our research, direct evidence indicated that Dip3 positively regulated the expression of *DOHH* and *DHYS*; this is a novel finding suggesting a new concept. Dip3 interaction with the viral protein Vank86, Vank92, and Vank101, inhibited eIF4E and eIF4E-dependent gene expression (41). Viral protein translation needs a specific translation system; in the MbBV system, the eIF4E-eIF4A protein translation system has been blocked (3, 47). Our data showed that *DOHH* and *DHYS* are eIF5A-denpendent genes, strongly supporting our hypothesis that integration mediated eIF5a hypusination. The virus triggers eIF5A activation, and when MbBV modifies hypusination, eIF5A translocates from the nucleus to promote its activation in the cytoplasm. Previous studies have reported that the HIV-1 Rev transactivator protein mediates the translocation of viral mRNAs from the nucleus to the cytoplasm, which is essential for expressing viral structural proteins (61). eIF5A specifically binds to Rev (62), and eIF5A loss-of-function mutants block the nuclear export of Rev protein and HIV1 replication (63). We hypothesized that the MbBV-mediated translocation of eIF5A from the nucleus to the cytoplasm promotes eIF5A activation by enabling eIF5A to carry viral proteins or host protein mRNA out of the nucleus, providing a large amount of substrate for protein translation.

The host cell with MbBV-mediated eIF5A hypusination drive apoptotic cells to generate ABs, containing MbBV virulence gene; these ABs are used to induce immunosuppression and alter the physiological and biochemical activities of cells (64, 65). Our data also showed that eIF5A-dependent host proteins, such as P53, CypD, CypA, and CypJ, affected cell apoptosis (66, 67). In related studies, the expression of virulence genes can be detected continuously in bracovirus-infected cell lines (38, 68). Therefore, we hypothesized that unlike proteins and mRNAs with a short half-life, DNA could be easily transmitted between infected and uninfected cells (69, 70).

Re-integration of MbBV fragments is a novel strategy indicating that the spread immunosuppression signaling may involve the homologous recombinant repair system. This is the first time that this strategy was demonstrated to be used in virus re-integration. Our findings demonstrate that MbBV DNA integration sets a foundation for viral gene transcription and plays an essential role in suppressing the host immune response at the DNA level. Moreover, MbBV DNAs separately integrate into different sites on different chromosomes, such as MdBV and CcBV (37, 38). However, the connection between viral DNA and immunosuppression remains nebulous. Researchers believe that DNA integration of PDVs is the basis of the expression of viral genes that function as immune suppressors and development inhibitors (54, 71). MbBV requires a structural foundation to complete its DNA integration, such as MdBV and CcBV (38). A comparative analysis of MbBV, MdBV, and CcBV genomes revealed that MbBV has HIMs on 15 of its 17 dsDNA circles. Although HIMs differ, they share similar structures, containing two boundary sites on both the 5` and 3` terminals, homologous junction 1 and junction 2 near boundary sites, and a non-homologous loop in the middle (38). We found that HIMs of MbBV harbor all these structures, and HIMs on Circle 14 and 16, and Scaffold F157 have the highest integration quantity. This phenomenon corroborates the findings from our previous report on the integration and expression of Vank86 on Circle 14 and Vank101 on Circle 16 (40). Previous studies have shown that the whole DNA circle of PDV can be integrated into the host genome (37, 38). In the ABs, MbBV fragments lost intact of HIM, and RAD51 recombinase, which repair double-stranded DNA breaks, mediated the re-integration.

Overall, our data provide a preliminary basis explaining how ABs participate in bracoviruses-mediated persistent immunosuppression. In addition, we found that MbBV can use ABs to escape immune surveillance. Whether other members of the PDV family can also transmit immunosuppressive signals between infected and uninfected cells through ABs warrants further studies.

## Methods Details

### Reagents

DHYS inhibitor GC7 was purchased from MedChemExpress (HY-108314A, MCE).

### Apoptosis Analysis

Analysis of apoptotic hemocytes was performed using an Annexin V-FITC/PI apoptosis detection kit (Vazyme, Nanjing, China) according to the manufacturer’s instructions.

### Insect Cultivation

The S. *litura* colony was grown on an artificial diet at 27 ± 1°C and 60%–80% humidity (72). The parasitoid *M. bicoloratus* colony was maintained on *S. litura* larvae grown in the laboratory. Adults were provided with honey as a dietary supplement (2).

### Cell Lines and Transfection

The *S. litura* (Spli221) (73), *S. frugiperda* (Sf9) (74), and cabbage looper *Trichoplusia ni* (High Five; provided by Sun Yat-Sen University) (75) cell lines were cultured at 27°C. All cell lines were cultured in TNM-FH insect culture medium containing 10% fetal bovine serum (HyClone; Cytiva, Marlborough, MA, USA), as previously described (58). Cells were transfected using a 4:1 ratio of X-trem Fugene Transfection Reagent (4 mL; Roche, Basel, Switzerland) and 1 mg DNA per well (1 mL) according to the manufacturer’s protocol. Transfection efficiencies ranged from 50% to 75% in different cell lines, as measured by GFP expression.

### Induction, Purification, and Isolation of ABs

Induction of ABs: 4 × 10^5^ Sf9 cells were placed in each well of a 6-well plate. After 2 h of attachment, four equivalent MbBV virus and three generations of reBac-Inx3 at 5% concentration were added. The culture was then continued for 72 h. In the control group, apoptosis was induced by UV irradiation, and the adherent cells were irradiated under a UV lamp (107 uw/cm^2^) for 2 h and then cultured for 70 h. The AB-induced cells were resuspended and placed into a centrifuge tube. They were centrifuged at 500 × *g* for 5 min at room temperature. The supernatant was transferred to a new centrifuge tube and then centrifuged at 3,000 × *g* for 20 min at room temperature. The supernatant was discarded, and the pellet was resuspended with 1× phosphate-buffered saline (PBS) and centrifuged at 3,000 × *g* for 20 min at room temperature. The above steps were repeated one more time. The precipitates were collected, and the ABs were resuspended in PBS and filtered using a 5-μm filter. The filtrate was centrifuged at 3,000 × *g* for 20 min at room temperature, and the precipitate was collected as ABs.

### Dilution of ABs

The ABs obtained by the above method were gently resuspended and mixed with 1 mL 10% bovine fetal serum medium. Several centrifuge tubes were taken, and different volumes of 10% fetal bovine serum medium (1.9, 1.8, 1.7, 1.4, and 1.2 mL) were added. In addition, 10% bovine fetal serum medium containing ABs was added into the corresponding centrifuge tubes (0.1, 0.2, 0.3, 0.6, and 0.8 mL) and gently mixed with a pipette gun for reserve use. To further confirm the biological role of ABs in recipient cells, we designed Abs of UV-irradiated Sf9 cells without biological and chemical pollution (76, 77) as a negative control.

### Scratch Test

In total, 2 × 10^5^ High Five cells were placed in each well of a 6-well plate and allowed to grow to cover the bottom of the 6-well plate. Using a 100 μL pipette, a cross line was marked at the bottom of the 6-well plate with the tip of a pipette. The ABs collected after different apoptotic induction treatments were added to the 6-well plate and observed and photographed under a microscope. After 24 h of growth, they were observed again under a microscope and photographed. Image J was used to calculate the wound healing area within 24 h.

### Characterization of reBac-Inx3-Induced AB

In a 6-well plate, 4 × 10^5^ Sf9 cells were placed in each well. Cells were infected with 5% reBac-Inx3 baculovirus of P3 generation for 72 h. The above cells were suspended, and the ABs were purified and collected by gradient centrifugation. The ABs were resuspended with 1 mL of 10% bovine fetal serum medium and mixed thoroughly. The ABs suspension (500 μL) was extracted for protein quantification. The untreated SF9 cells were incubated with another 500 μL of the AB solution for 72 h. After incubation, the cells were washed twice with PBS and then collected for protein quantification.

### Hemocytes Isolation

Hemolymph (500 μL) isolated from ∼100 larvae of *S. litura* was centrifuged at 4°C for 5 min at 1,000 × *g*, and pellets were collected as hemocytes. The pellets were centrifuged for 5 min at 1000 × *g*, washed with 1× PBS twice, and resuspended in 1 mL PBS. Hemocytes isolated from parasitized larvae were referred to as parasitized samples, and those from unparasitized larvae were used as controls (78).

### Genome Sequencing and Analysis

The samples were sequenced using an Illumina Hiseq 2000, and the total number of bases sequenced was greater than 3 Gbp. *de novo* DNA-seq assembly was performed using BWA, Velvet and ABySS software (79–81). The viral DNA sequences contained in the total DNA-seq were assembled by using SOAPdenovo (82), Platanus (83) and Cap3 (84). GeneMark was used to identify the functional proteins from the isolated contigs (85). Genome sequences were used to blast with genome of MbBV (40) and genome of normal *S. litura* larvae (39) by using Blast (v 2.6.0+) (86).

### MbBV Isolation and Cell Infection

MbBV viral particles were purified as previously described (49). Briefly, fresh wasps were frozen at -20°C for 10 min and then placed on ice. The reproductive tracts of the female wasps were excised under a binocular stereomicroscope, and the separated ovaries were collected into a 1.5-mL Eppendorf tube on ice. The calyces were then punctured using forceps, the calyx fluid was resuspended in 1× PBS, and the resuspension was ground using a 2.5-mL syringe. The mixture was centrifuged for 3 min at 1,000 × *g* at 4°C to remove the eggs and cellular debris. A 0.45-μm syringe filter was used to purify the virions. Spli221 cells (1.5 × 10^5^) were seeded in a 12-well culture plate (Corning Inc., Corning, NY, USA) 2 h before infection. The virions from one wasp could infect 1 × 10^5^ Spli221 cells. Purified virions were added to each well in 1 × 10^5^ cells/one wasp-derived MbBV equivalents, as previously described.

### Apoptosis Analysis of Hemocytes

Briefly, after seven days of RNAi feeding, hemocytes (1 × 10^5^) were extracted from *S. litura* larvae (n = 3), resuspended in 100 μL of 1× binding buffer, and incubated with 5 μL of Annexin V-FITC dye and 5 μL of PI dye for 10 min on ice in the dark. After adding 400 μL of 1× binding buffer, an Olympus IX71 inverted fluorescence microscope with FV10-ASW 4.0 Viewer software (Olympus) was used to detect the apoptotic hemocytes. Early apoptotic cells identified by Annexin V-FITC showed green fluorescence, while late apoptotic cells stained by PI showed both green and red fluorescence. Five fields of more than 100 cells within each well of a 12-well plate were captured for analysis and quantification; experiments were performed in triplicate, and we obtained 15 images (from three wells) for each treatment for visual inspection and data quantification.

### Total DNA Extraction

Total DNA was isolated from hemocytes and Spli221 cells. Cells (2×10^6^) were incubated in 200 μL of lysis buffer (100 mM NaCl, 10 mM Tris/HCl, 25 mM EDTA, 0.5% SDS, pH 8.0) containing 2.5 mg of proteinase K per mL, 8 μL of 20% Sarcosyl solution, and 1 mg of RNaseA/mL at 55°C for 5 h. The isolated DNA was further purified by phenol-chloroform extraction and subsequent ethanol precipitation. The concentration of each DNA sample was determined by measuring the optical density at A260/A280 using a NanoDrop 2000 and using 1×TBE agarose gels. DNA was prepared for sequencing and further PCR amplification. High quality samples (with an A260/A280 ratio ≥ 2.0, A_260_/A_230_ ≥ 2.0, concentration ≥ 500 ng/μL) were stored at −20°C until use. DNA was prepared from at least three biological replicates.

### Proteomics of Parasitized Hemocytes and MbBV Infected Spli221 Cells

MbBV infected Spli221 cells were isolated, and protein sequences, for gene expression were determined using tandem mass spectrometry to examine the expression of genes of the parasitized hemocytes.

### Construction of eIF5A, DHYS, and DOHH Expression Plasmids

For eIF5A, DHYS and DOHH overexpression, the corresponding genes were amplified by PCR using cDNA as a template and the following primers: E-*eIF5A*-F (5′-GAA TTC ATG GCT GAT ATC GAG GA-3′) and E-*eIF5A*-R (5′-GCG GCC GCA TTT GTC AA-3′), containing *EcoR*I and *Not*I sites (underlined); E-*DHYS*-F (5′-GGT ACC ATG GAT ATA ACT TCA GCT A-3′) and E-*dip3*-R (5′-TCT AGA TAA ACA TTC TTT TTA TTG C-3′), containing *Kpn*I and *Xba*I sites (underlined), and E-*DOHH*-F (5′-GAG CTC ATG GCA AAA GCT AG-3′) and E-*DOHH*-R (5′-TCT AGA CAG CCC TCG ACA GT-3′), containing *Sac*I and *Xba*I sites (underlined).The genes were directionally cloned into the pMD19 vector (Takara Bio, Kusatsu, Japan), and the inserts were confirmed by direct sequencing. Finally, the eukaryotic expression plasmid pIZT/V5-His (Invitrogen, Carlsbad, CA, USA) was used for the expression of eIF5A, DHYA and DOHH fusion proteins with V5 and 6 × His tags.

### Antibodies and Western Blotting

Western blotting was performed as described (58). The following primary antibodies were used: mouse anti-V5 (1:5,000; Thermo Fisher Scientific, Waltham, MA, USA), mouse anti-tubulin (1:2,000; Solarbio, Beijing, China), rabbit anti-GAPDH (1:2,000; Solarbio), and rabbit anti-Hypusine (EMD Millipore; 1:1,000) antibodies, rabbit anti-eIF5A (1:1,000; ABclonal), rabbit anti-DHPS (1:200; abcam), rabbit anti-DOHH (1:500; Sigma-Aldrich). Secondary antibodies used were goat anti-mouse horseradish peroxidase-conjugated secondary antibody (1:2,000; Beyotime, Shanghai, China) and goat anti-rabbit horseradish peroxidase-conjugated secondary antibody (1:5,000). Anti-His (M1001020, Solarbio), Anti-pAKT473 (GB13012-3, Servicebio), Anti-pAKT308 (ab66134, Abcam), Anti-ATPaseβ (GL Binchem synthesis), Anti-Inx1(GL Binchem synthesis), Anti-Inx2 (GL Binchem synthesis), Anti-Inx3(GL Binchem synthesis), Anti-Inx4 (GL Binchem synthesis), Cleaved-caspase (WL02117, Wanleibio), Anti-GAPDH (M1000110, Solarbio), Anti-β Tubulin (AF1216, Beyotime), Goat Anti-Mouse (A0216, Beyotime), Goat Anti-Rabbit (A0208, Beyotime). Proteins were semi-quantified *via* densitometry using ImageJ (National Institutes of Health, Bethesda, MD, USA).

### Electrophoretic Mobility Shift Assay (EMSA) and Nonlinear Regression Curve and Kd Value Determination

The promoter prediction, the eukaryotic protein expression, and EMSA were performed as described (87–89). First, we used the website http://gene-regulation.com to analyze the domain binding of the promoter region sequences of DOHH and DHYS. We screened out the gene sequences that may interact with the myb/SANT domain of Dip3. Then, through the designed primers, the target gene was obtained by PCR from the whole genome of *S. litura* hemolymph. Subsequently, we sonicated the BL21(DE3) *Escherichia coli* carrying pET28a His-Dip3 and pET28a His-Vank86 prokaryotic expression, and vector and extracted the crude protein samples of His-Dip3 and His-Vank86. We then immediately purified the crude protein samples obtained above by nickel magnetic beads to obtain purified His-Dip3 and His-Vank86 samples. Finally, we took the purified His-Dip3 and His-Vank86 proteins, set four gradients (0, 5, 10, and 20 μg), added 1 μg of target DNA and 10×Binding Buffer, and incubated the mixture at room temperature for 30 min. The samples were run on (100 V 30 min) agarose gel, to observe the results.

Nonlinear regression curve and K_d_ value determination were analyzed as described (90, 91). We used the ImageJ program to analyze the agarose gel image by inverting the image first, and then use ImageJ to measure the raw integrated density (RID) of each band. The results were exported to an Excel table to perform the following calculations. Each RID value was subtracted from the lowest RID value, and the lowest RID value was subtracted from itself, and the result was zero. These values are called relative RIDs. Each relative RID value was divided by the largest relative RID and then multiplied by 100. The maximum relative RID value was divided by itself, multiplied by 100, and the result was 100. These values are called percentage RID values. Finally, the percentage RID value was subtracted from 100 to get the binding rate. The obtained binding rate was analyzed using GraphPad Prism. An XY list was created, the protein concentration being in the X list, and the binding rate being in the Y list. The analysis option was selected, the equation labeled “a site-specific binding” was chosen, and the K_d_ value was calculated to get a nonlinear regression line.

### Immunofluorescence

Immunofluorescence was performed as previously described (58), with minor modifications. High Five Cells grown on coverslips were fixed in 4% paraformaldehyde for 15 min and permeabilized in 0.2% Triton X-100 in PBS (PBST). The fixed cells were blocked with 5% normal goat serum in 0.1% PBST for 15 min. Ectopically expressed V5-fused eIF5A were identified using a mouse anti-V5 antibody (1:2,000; Thermo Fisher Scientific) and AlexaFluor^®^568 Goat anti-mouse IgG (H+L) (1:2,000; Thermo Fisher Scientific). Labeled cells were incubated with phalloidin (Sigma-Aldrich, St. Louis, MO, USA) diluted 1:40 in PBS for 1 h at 37°C. Cells were then washed with PBS and incubated with 4, 6-diamidino-2-phenylindole (DAPI; 1:1,000; Roche) for 5 min. Slides were mounted with mounting medium, antifading (Cat. No. S2100; Solarbio). Cells were imaged using Confocal (ZEISS LSM 800) microscopy.

### Plasmid Construction for dsRNA Feeding

Plasmids were constructed as described previously (92). The sequences encoding the *eIF5A*, *DHYS* and *DOHH* genes were inserted into the RNAi vector L4440, containing two convergent T7 polymerase promoters in opposite orientation separated by a multiple cloning site.

### Preparation of dsRNA and dsRNA Feeding

The plasmids for dsRNA feeding were transformed into the bacterial host *E. coli* HT115 (DE3). In brief, a single colony of HT115 containing the recombinant L4440 vector was inoculated in 4 mL of LB medium containing 4 μL ampicillin (100 μg/mL) and 4 μL tetracycline (100 μg/mL) and cultured overnight at 37°C. The cultures were diluted to reach an OD_600_ of 0.4. Isopropyl-β-D-thiogalactopyranoside (IPTG) was then added at a final concentration of 0.8 mM, and the cultures were incubated for 4 h with shaking at 37°C. An aliquot of the suspension (200 mL) was centrifuged at 10,000 × *g* for 10 min; the pellet (OD_600_ ≈ 1) was resuspended in 5 mL of sterile H_2_O after cooling to 50°C and mixed with 50 mL of freshly prepared artificial feed. The prepared dsRNA feed was stored in a small plastic box (9.5 × 7.0 × 5.5 cm, 200 mL) at 4°C and used within one week. Control *S. litura* received a standard feed. Before hatching, *S. litura* eggs were placed on artificial food. For long-term maintenance of the dsRNA diet, larvae were provided with fresh food every day (47). Hemocytes were harvested on day 7 for apoptosis assays. Head capsule measurement was performed daily. EGFP dsRNA was used as control dsRNA.

### RT-qPCR

The recipient cells incubated with ABs were collected. Total RNA was isolated from the samples using the RNAiso Plus kit (Takara Bio) and then treated with DNA enzymes. NanoDrop 2000 was used to measure the concentration and OD_260_/OD_280_ of each RNA sample. cDNA was then synthesized by PrimeScript II 1st Strand cDNA Synthesis Kit (Takara Bio). All cDNA samples were stored at -80°C. The above-mentioned synthesized cDNA was used as a template for RT-qPCR analysis. The primers used were: Q-vank86-F(5’- CTC AGA CGG CGT TCA-3’),Q-vank86-R(5’- TCG CAG TAG CCA GAC A -3’); Q-vank92-F(5’- CCT CTG CCG TGA TG-3’),Q-vank92-R(5’- CGA AAA CTC GCT CTT G -3’); Q-vank101-F(5’- CCT TAG ACT GGG AGC GAC AT-3’), Q-vank101-R(5’- ACG CTG CTT CGT GGA GG-3’); Q-18S-F(5’-CTG ATT CCC CGT TAC CCG TGA-3’),Q-18S-R(5’-AGA ACT CTG ACC AGT GAT GGG ATG-3’). The latter two primers were used to amplify 18S as endogenous controls. Cycle parameters are as follows: 95°C for 30 s; 40 cycles of 95°C for 5 s; 40 cycles of 60°C for 34 s; 95°C for 15 s; 60°C for 1 min; 95°C for 15 s and an indefinite hold at 10°C. Each sample was repeated for 3 times. The relative gene expression was calculated by 2^-ΔΔCT^ method.

### Quantification and Statistical Analysis

Data were analyzed using GraphPad Prism (ver. 7, Prism), and statistical significance was determined using the Student’s *t*-test for unpaired experiments (two-tailed). *p* < 0.05 indicates statistically significant difference between groups. The resulting data are presented as means ± SEM from at least three independent experiments.

## Data Availability Statement

The datasets presented in this study can be found in online repositories. The names of the repository/repositories and accession number(s) can be found below: https://doi.org/10.6084/m9.figshare.19430468.v1 and https://doi.org/10.6084/m9.figshare.19443284.v1.

## Author Contributions

K-JL conceived and supervised the study. G-FZ and XY determined eIF5A hypusination assays. C-XC and N-NP prepared ABs and perform ABs transmitting immunosuppressive assays. Q-CC and X-CL performed MbBV integration assays and genome sequencing and analysis. J-HC and Q-CC performed proteomics assays. Conceptualization, K-JL; Methodology, K-JL, G-FZ, C-XC, Q-CC, and J-HC; Investigation, G-FZ, C-XC, Q-CC, XY, N-NP, X-CL, J-HC, Y-FH, QZ, J-HM, C-HC, and H-MT; Writing-original draft, G-FZ, C-XC, Q-CC, XY, and N-NP; Writing-review & editing, K-JL, G-FZ, XY, N-NP; Funding acquisition, K-JL. All authors contributed to the article and approved the submitted version.

## Funding

This work was supported by the Science and Technology Planning Project in Key Areas of Yunnan Province [grant number 202001BB050002], National Natural Science Foundation of China [grant numbers 32160662, 31772225, 31471823, 31260448, and 31060251], NSFC-NRF [grant number 31411140238 to K-JL], and Yunnan Department of Science and Technology [grant number 2013FA003 to K-JL]. K-JL was supported by the Donglu Scholar Program of Yunnan University.

## Conflict of Interest

The authors declare that the research was conducted in the absence of any commercial or financial relationships that could be construed as a potential conflict of interest.

## Publisher’s Note

All claims expressed in this article are solely those of the authors and do not necessarily represent those of their affiliated organizations, or those of the publisher, the editors and the reviewers. Any product that may be evaluated in this article, or claim that may be made by its manufacturer, is not guaranteed or endorsed by the publisher.

## Supplementary Material

Supplementary Figure 1 Flowchart showing the generation of apoptotic bodies, Related to **Figure 1**. **(A)** Structure model of N-terminal extended Inx3. **(B)** Schematic illustration of the flow of apoptotic bodies equipartition experiment. **(C)** Method of creating dilution gradient of apoptotic bodies. **(D)** Apoptosis detection of recipient Sf9 cells incubated with apoptotic bodies induced by UV, MbBV and re-Bac-Inx3, Scale bar, 20 μm.

Supplementary Figure 2 Bracovirus integrates host *Spodoptera litura* genome *via* host integrated motif, Related to **Figure 2**. **(A)** Schematic illustration of analysis of the host genome integrated by MbBV. **(B)** Host integration sites (HIMs) of MbBV genome. MbBV has 17 HIMs, 15 of which are located in the dsDNA circle, while the remaining 2 appear in short scaffolds. All HIMs have 2 Boundary sites (BS) and 2 Junction sites (JS). **(C)** Sequence of HIM JS 1 and JS 2 used to scan *S. litura* chromosomes. **(D)** The integrated DNA scaffold sequences detected. The primers designed to amplify HIM in the chromosome1, 9, 16, and 19 of *S. litura* (top); HIM of MbBV in circles 10, 11, 14, and 16 are detected with PCR, 6-days post-parasitism and 72 hours post infection by MbBV (mid), *vanks* and *PTP* genes detected in the DNA of parasitism hemocytes (bot).

Supplementary Figure 3 Proteins involved in eIF5A hypusination translation, Related to **Figure 3**. **(A)** Method of sample collection from hemocytes used MS/MS. **(B)** Identified proteins. **(C)** Methods of iTRAQ and transcription analysis. **(D)** Percentages of proline, glycine and charged amino acid in the up-regulated and down-regulated proteins in 6-day p.p hemocytes was analyzed *via* protein sequence.

Supplementary Figure 4 Bracovirus integrated viral genes dependent eIF5A hypusination translation pattern, Related to **Figure 4**. **(A)** Schematic illustration of the predicted promoter sequence of DHYS in Chromosome 26 of *S. litura*. **(B)** Schematic illustration of the predicted promoter sequence of DOHH in Chromosome 29 of *S. litura*.

Supplementary Figure 5 MbBV activated hypusination pathway *via* promoting eIF5A the nucleocytoplasmic transport, Related to **Figure 5**. **(A, B)** Ectopic expression of eIF5A, DHYS, and DOHH cannot modify hypusionation in High Five cells. Hypusine-dependent proteins were detected using western blotting. * *p*<0.05, ns, no significant difference, error bars represent SEM. Unpaired Student’s *t*-test with Holm-Sidak method for multiple *t* test; n = 3. **(C)** Immunofluorescence was used to detect location of DHYS and DOHH in MbBV infected High Five cells. Scale bar, 20 μm.

Supplementary Figure 6 Bracovirus integration-mediated eIF5A hypusination drives persistence apoptosis, Related to **Figure 6**. **(A)** Effect of *eIF5A* dsRNA on the width of the head shell of *S. litura*. **(B)** Effect of the silencing *eIF5A* after parasitism on the width of the head shell of *S. litura*. **(C)** Effect of *DHYS* dsRNA on the width of the head shell of *S. litura*. **(D)** Effect of the silencing *DHYS* after parasitism on the width of the head shell of *S. litura*. **(E)** Effect of *DOHH* dsRNA on the width of the head shell of *S. litura*. **(F)** Effect of the silencing *DOHH* after parasitism on the width of the head shell of *S. litura*. **p*<0.05, ***p*<0.01, ****p*<0.001, *****p*<0.0001, ns, no significant difference, error bars represent SEM. Unpaired Student’s *t*-test with Holm-Sidak method for multiple *t* test; n = 3.

Supplementary Table 1 Location of HIM on MbBV genome (Related to **Figure 2**).

Supplementary Table 2 Sequencing scaffold with detected MbBV integration events (Related to **Figure 2**).

Supplementary Table 3 Rich in proline glycine and charged amino acid proteins in Spodoptera litura hemocytes parasitized by Microplitis bicoloratus (Related to **Figure 4**).
