# Supplementary figures and images for: Bracovirus Sneaks Into Apoptotic Bodies Transmitting Immunosuppressive Signaling Driven by Integration-Mediated eIF5A Hypusination
Source: Front Immunol. 2022 May 17;13:901593. doi: 10.3389/fimmu.2022.901593 (PMC9156803; doi:10.3389/fimmu.2022.901593)

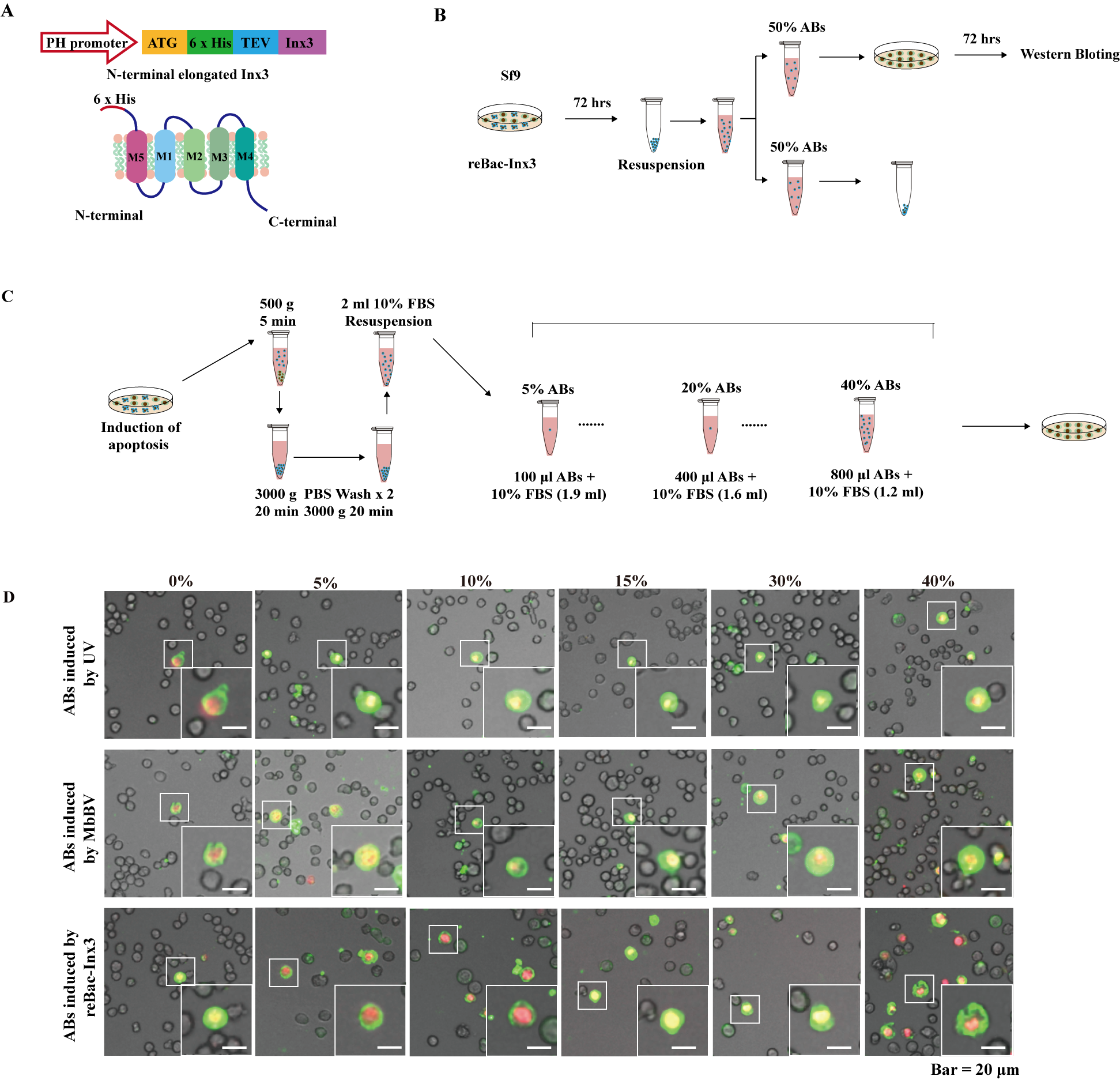

Supplement: Supplementary Figure 1 — Flowchart showing the generation of apoptotic bodies, Related to Figure 1 . (A) Structure model of N-terminal extended Inx3. (B) Schematic illustration of the flow of apoptotic bodies equipartition experiment. (C) Method of creating dilution gradient of apoptotic bodies. (D) Apoptosis detection of recipient Sf9 cells incubated with apoptotic bodies induced by UV, MbBV and re-Bac-Inx3, Scale bar, 20 μm. [file Image_1.tif]

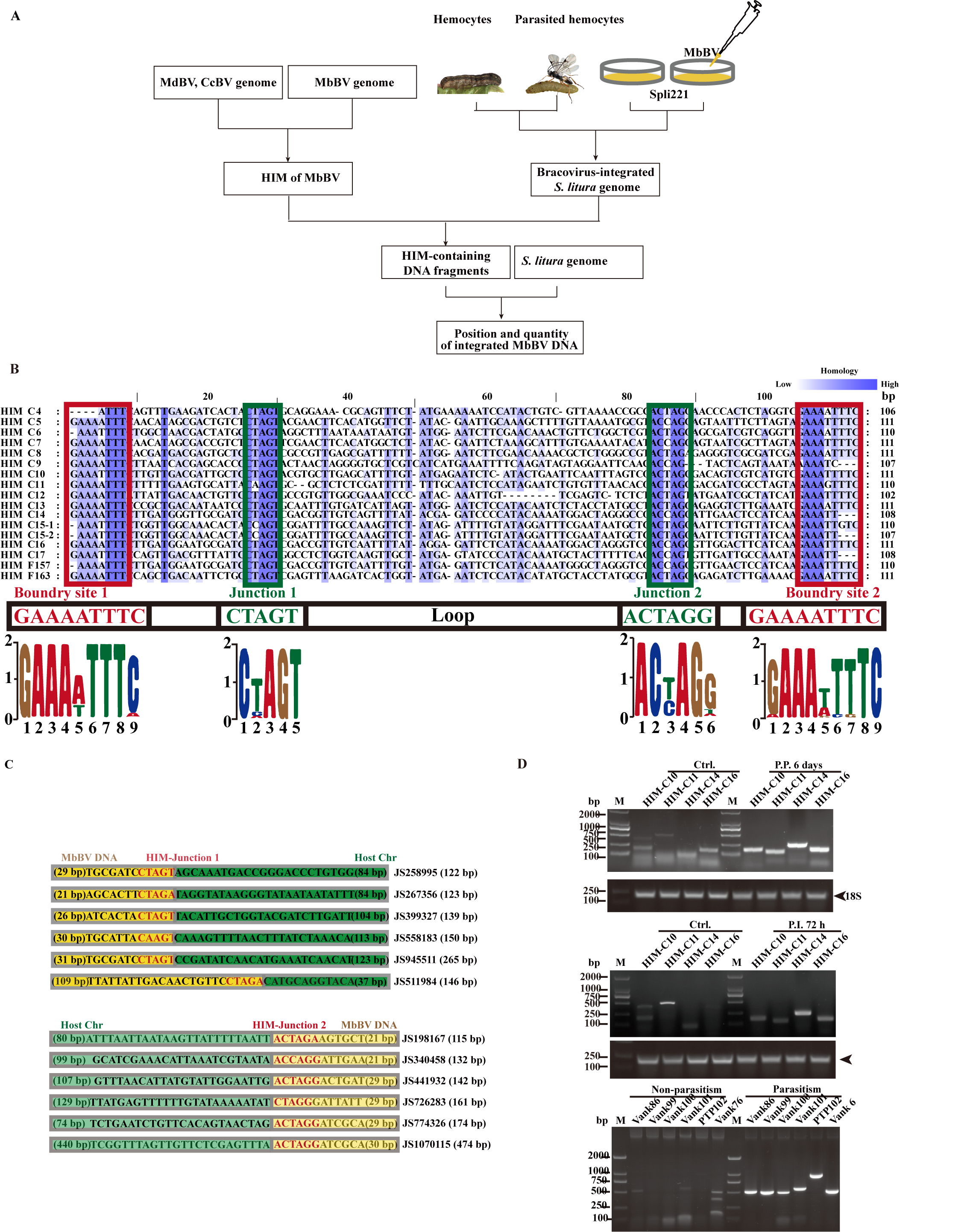

Supplement: Supplementary Figure 2 — Bracovirus integrates host Spodoptera litura genome via host integrated motif, Related to Figure 2 . (A) Schematic illustration of analysis of the host genome integrated by MbBV. (B) Host integration sites (HIMs) of MbBV genome. MbBV has 17 HIMs, 15 of which are located in the dsDNA circle, while the remaining 2 appear in short scaffolds. All HIMs have 2 Boundary sites (BS) and 2 Junction sites (JS). (C) Sequence of HIM JS 1 and JS 2 used to scan S. litura chromosomes. (D) The integrated DNA scaffold sequences detected. The primers designed to amplify HIM in the chromosome1, 9, 16, and 19 of S. litura (top); HIM of MbBV in circles 10, 11, 14, and 16 are detected with PCR, 6-days post-parasitism and 72 hours post infection by MbBV (mid), vanks and PTP genes detected in the DNA of parasitism hemocytes (bot). [file Image_2.tif]

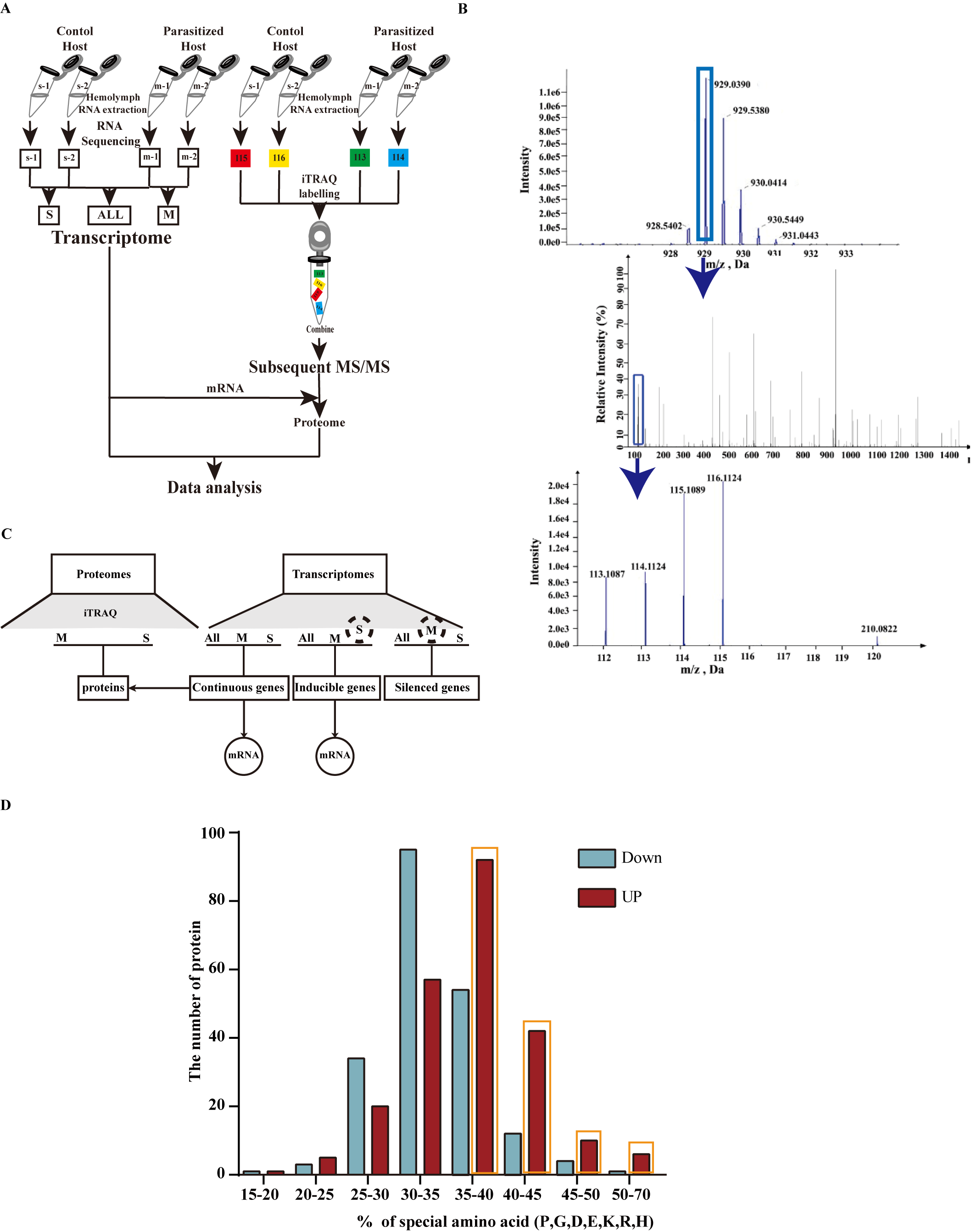

Supplement: Supplementary Figure 3 — Proteins involved in eIF5A hypusination translation, Related to Figure 3 . (A) Method of sample collection from hemocytes used MS/MS. (B) Identified proteins. (C) Methods of iTRAQ and transcription analysis. (D) Percentages of proline, glycine and charged amino acid in the up-regulated and down-regulated proteins in 6-day p.p hemocytes was analyzed via protein sequence. [file Image_3.tif]

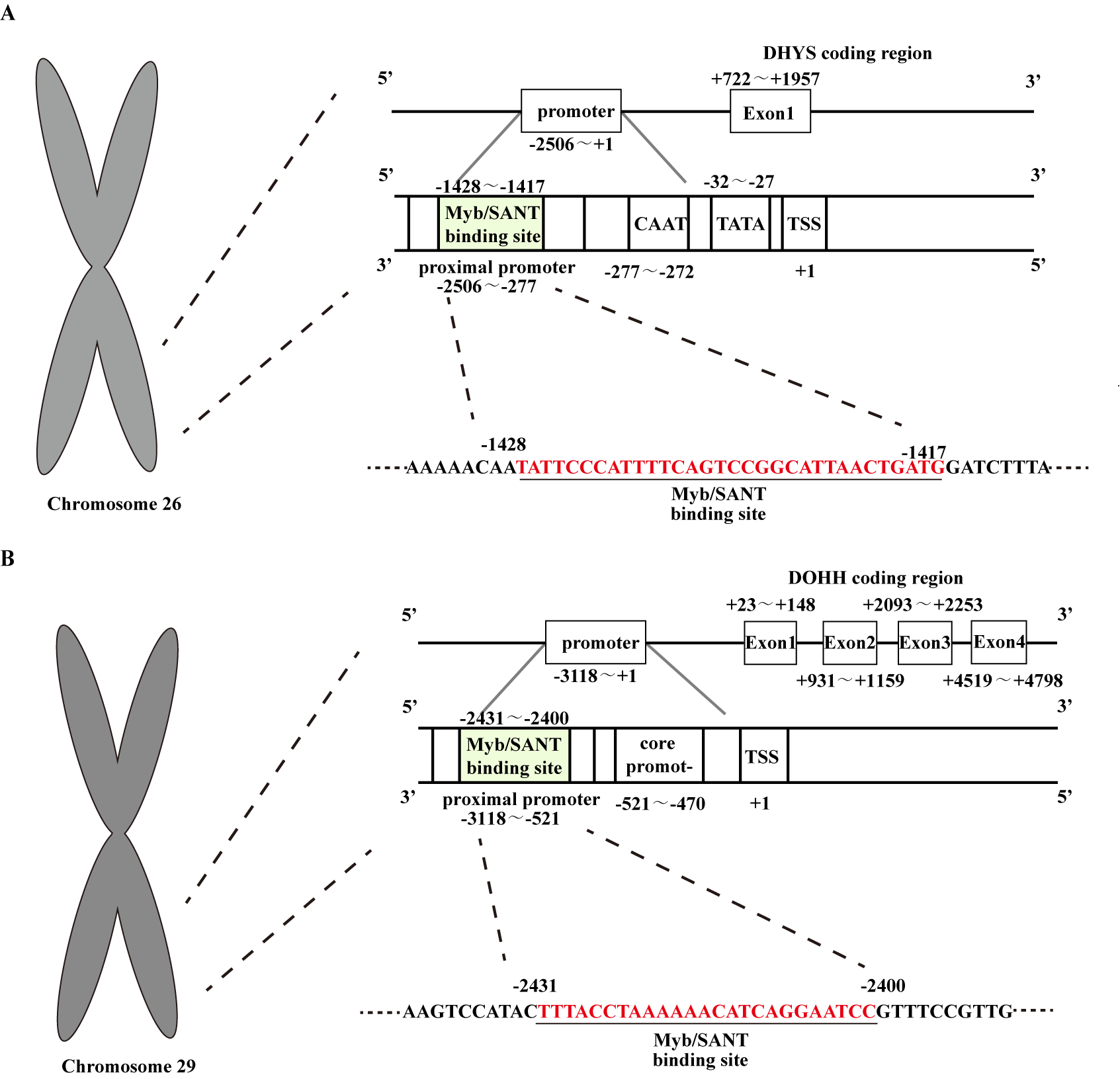

Supplement: Supplementary Figure 4 — Bracovirus integrated viral genes dependent eIF5A hypusination translation pattern, Related to Figure 4 . (A) Schematic illustration of the predicted promoter sequence of DHYS in Chromosome 26 of S. litura. (B) Schematic illustration of the predicted promoter sequence of DOHH in Chromosome 29 of S. litura. [file Image_4.tif]

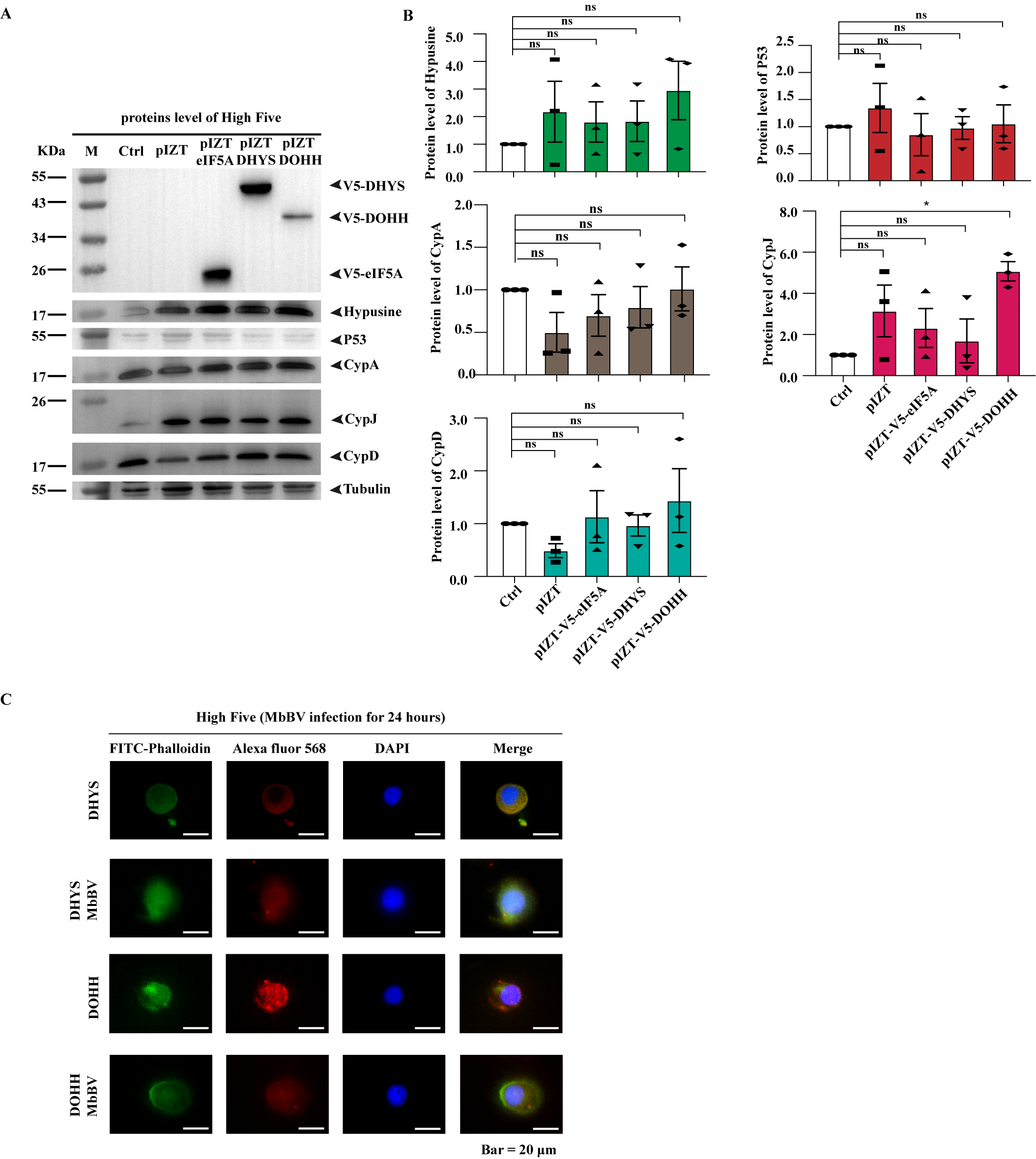

Supplement: Supplementary Figure 5 — MbBV activated hypusination pathway via promoting eIF5A the nucleocytoplasmic transport, Related to Figure 5 . (A, B) Ectopic expression of eIF5A, DHYS, and DOHH cannot modify hypusionation in High Five cells. Hypusine-dependent proteins were detected using western blotting. * p<0.05, ns, no significant difference, error bars represent SEM. Unpaired Student’s t-test with Holm-Sidak method for multiple t test; n = 3. (C) Immunofluorescence was used to detect location of DHYS and DOHH in MbBV infected High Five cells. Scale bar, 20 μm. [file Image_5.tif]

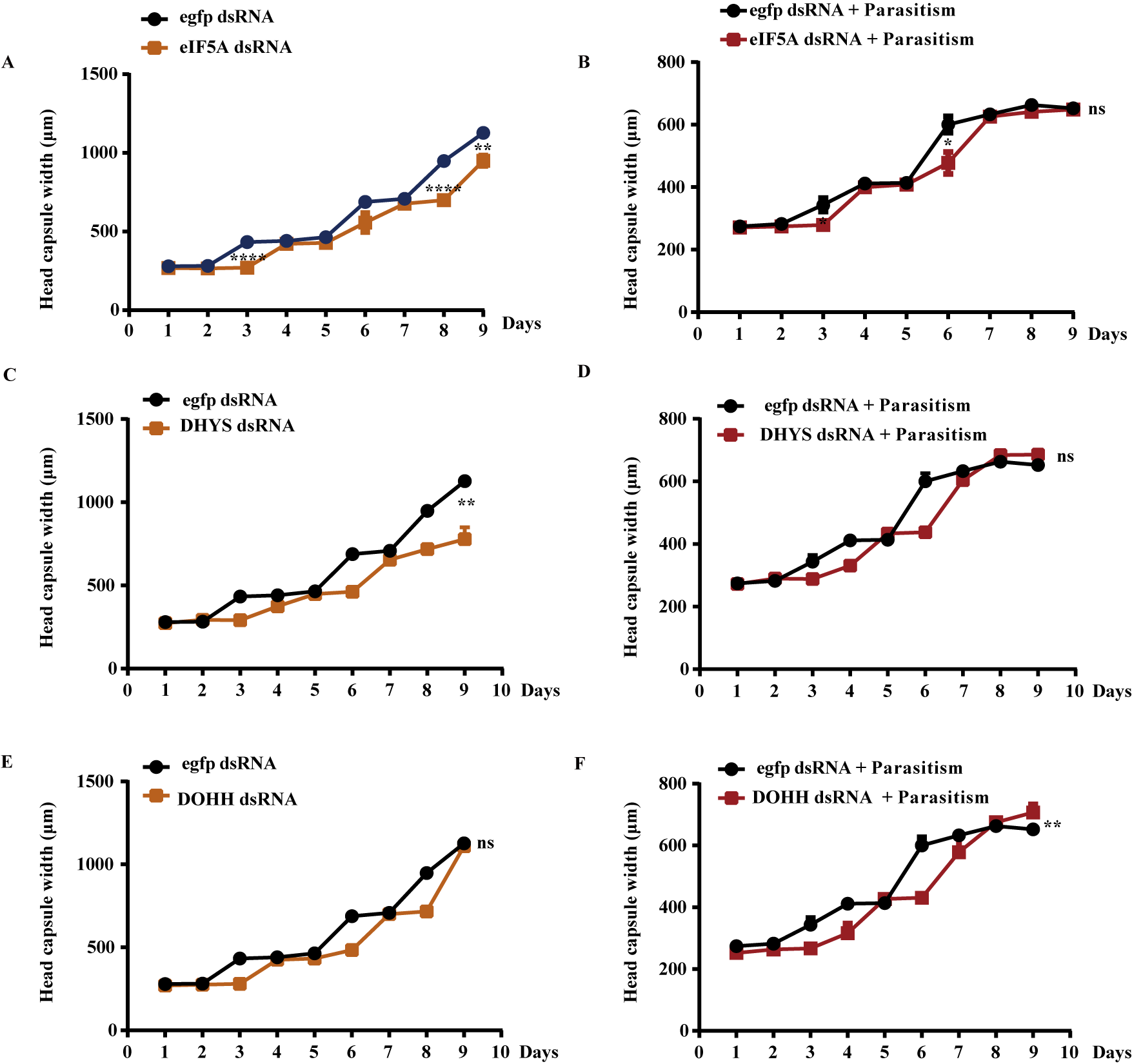

Supplement: Supplementary Figure 6 — Bracovirus integration-mediated eIF5A hypusination drives persistence apoptosis, Related to Figure 6 . (A) Effect of eIF5A dsRNA on the width of the head shell of S. litura. (B) Effect of the silencing eIF5A after parasitism on the width of the head shell of S. litura. (C) Effect of DHYS dsRNA on the width of the head shell of S. litura. (D) Effect of the silencing DHYS after parasitism on the width of the head shell of S. litura. (E) Effect of DOHH dsRNA on the width of the head shell of S. litura. (F) Effect of the silencing DOHH after parasitism on the width of the head shell of S. litura. *p<0.05, **p<0.01, ***p<0.001, ****p<0.0001, ns, no significant difference, error bars represent SEM. Unpaired Student’s t-test with Holm-Sidak method for multiple t test; n = 3. [file Image_6.tif]
