# Supplementary material for: Bracovirus Sneaks Into Apoptotic Bodies Transmitting Immunosuppressive Signaling Driven by Integration-Mediated eIF5A Hypusination
Source: Front Immunol. 2022 May 17;13:901593. doi: 10.3389/fimmu.2022.901593 (PMC9156803; doi:10.3389/fimmu.2022.901593)
Supplement: Supplementary Table 1 — Location of HIM on MbBV genome (Related to Figure 2 ). [file Table_1.docx]

| **Table S1 Location of HIM on MbBV genome** (Related to FIGURE 2)   \| Name \| Length（bp） \| Contig \| Contig start \| Contig end \| Identity（%） \| E value \| Reference \| Ref start \| Ref end \| Identity（%） \| E value \| \| --- \| --- \| --- \| --- \| --- \| --- \| --- \| --- \| --- \| --- \| --- \| --- \| \| HIM C4 \| 106 \| Node 169 \| 675 \| 780 \| 100 \| 1.00E-56 \| HIM E \| 5 \| 111 \| 89.72 \| 1.00E-30 \| \| HIM C5 \| 111 \| Node 118 \| 101 \| 211 \| 100 \| 1.00E-59 \| HIM A \| 1 \| 111 \| 92.79 \| 1.00E-42 \| \| HIM C6 \| 110 \| Node62 \| 530 \| 639 \| 100 \| 5.00E-59 \| HIM C \| 2 \| 111 \| 94.55 \| 8.00E-47 \| \| HIM C7 \| 111 \| Node 36 \| 1112 \| 1222 \| 100 \| 1.00E-59 \| HIM B \| 1 \| 111 \| 89.19 \| 4.00E-33 \| \| HIM C8 \| 111 \| Node 8 \| 1904 \| 2014 \| 100 \| 1.00E-59 \| HIM F \| 1 \| 111 \| 91.89 \| 3.00E-40 \| \| HIM C9 \| 107 \| Node 21 \| 1461 \| 1355 \| 100 \| 3.00E-57 \| HIM G \| 1 \| 32 \| 90.63 \| 2.00E-07 \| \| HIM C10 \| 111 \| Node 34 \| 1978 \| 2088 \| 100 \| 1.00E-59 \| HIM I \| 1 \| 111 \| 90.99 \| 7.00E-38 \| \| HIM C11 \| 110 \| Node 65 \| 5159 \| 5304 \| 100 \| 5.00E-59 \| HIM H \| 1 \| 110 \| 90 \| 7.00E-35 \| \| HIM C12 \| 102 \| Node 45 \| 3399 \| 3510 \| 100 \| 2.00E-39 \| HIM M \| 1 \| 102 \| 85.29 \| 1.00E-20 \| \| HIM C13 \| 111 \| Node 22 \| 20 \| 130 \| 100 \| 1.00E-59 \| HIM K1 \| 1 \| 111 \| 86.49 \| 6.00E-26 \| \| Node 83 \| 3360 \| 3420 \| 100 \| 1.00E-59 \| HIM K2 \| 1 \| 111 \| 86.49 \| 6.00E-26 \| \| HIM C14 \| 108 \| Node 25 \| 2614 \| 2721 \| 100 \| 8.00E-58 \| HIM J \| 1 \| 107 \| 87.96 \| 1.00E-26 \| \| HIM C15-1 \| 110 \| Node 13 \| 14434 \| 14543 \| 100 \| 5.00E-59 \| HIM L \| 2 \| 108 \| 84.11 \| 9.00E-19 \| \| HIM C15-2 \| 107 \| Node 13 \| 14437 \| 14543 \| 100 \| 3.00E-57 \| HIM L \| 1 \| 108 \| 84.11 \| 8.00E-19 \| \| HIM C16 \| 111 \| Node 18 \| 5491 \| 5601 \| 100 \| 1.00E-59 \| HIM N \| 1 \| 111 \| 98.2 \| 6.00E-57 \| \| HIM C17 \| 108 \| JS1091994 \| 147 \| 254 \| 100 \| 9.00E-55 \| HIM D \| 1 \| 108 \| 89.81 \| 1.00E-33 \| \| HIM F157 \| 110 \| Node 92 \| 1032 \| 1141 \| 100 \| 5.00E-59 \| HIM N \| 2 \| 111 \| 94.55 \| 8.00E-47 \| \| HIM F163 \| 111 \| Node 7 \| 1621 \| 1731 \| 100 \| 1.00E-59 \| HIM K1 \| 1 \| 111 \| 89.19 \| 4.00E-33 \| |
| --- | --- | --- | --- | --- | --- | --- | --- | --- | --- | --- | --- | --- | --- | --- | --- | --- | --- | --- | --- | --- | --- | --- | --- | --- | --- | --- | --- | --- | --- | --- | --- | --- | --- | --- | --- | --- | --- | --- | --- | --- | --- | --- | --- | --- | --- | --- | --- | --- | --- | --- | --- | --- | --- | --- | --- | --- | --- | --- | --- | --- | --- | --- | --- | --- | --- | --- | --- | --- | --- | --- | --- | --- | --- | --- | --- | --- | --- | --- | --- | --- | --- | --- | --- | --- | --- | --- | --- | --- | --- | --- | --- | --- | --- | --- | --- | --- | --- | --- | --- | --- | --- | --- | --- | --- | --- | --- | --- | --- | --- | --- | --- | --- | --- | --- | --- | --- | --- | --- | --- | --- | --- | --- | --- | --- | --- | --- | --- | --- | --- | --- | --- | --- | --- | --- | --- | --- | --- | --- | --- | --- | --- | --- | --- | --- | --- | --- | --- | --- | --- | --- | --- | --- | --- | --- | --- | --- | --- | --- | --- | --- | --- | --- | --- | --- | --- | --- | --- | --- | --- | --- | --- | --- | --- | --- | --- | --- | --- | --- | --- | --- | --- | --- | --- | --- | --- | --- | --- | --- | --- | --- | --- | --- | --- | --- | --- | --- | --- | --- | --- | --- | --- | --- | --- | --- | --- | --- | --- | --- | --- | --- | --- | --- | --- | --- | --- | --- | --- | --- | --- | --- | --- | --- | --- | --- | --- | --- |
