# Supplementary material for: Bracovirus Sneaks Into Apoptotic Bodies Transmitting Immunosuppressive Signaling Driven by Integration-Mediated eIF5A Hypusination
Source: Front Immunol. 2022 May 17;13:901593. doi: 10.3389/fimmu.2022.901593 (PMC9156803; doi:10.3389/fimmu.2022.901593)
Supplement: Supplementary Table 2 — Sequencing scaffold with detected MbBV integration events (Related to Figure 2 ). [file Table_2.docx]

| **Table S2 Sequencing scaffold with detected MbBV integration events** (Related to FIGURE 2)   \| Scaffold  name \| Length (bp) \| Align  length (bp) \| Chr  location \| Start  position \| End  position \| Scaffold  Indentity(%) \| Scaffold  E-value \| Integrated  MbBV HIM \| Integrated  Length (bp) \| HIM  Indentity(%) \| HIM  E-value \| \| --- \| --- \| --- \| --- \| --- \| --- \| --- \| --- \| --- \| --- \| --- \| --- \| \| JS198167 \| 115 \| 80 \| Chr 15 \| 2327116 \| 2327195 \| 100 \| 6.00E-38 \| HIM-C15 \| 21 \| 100 \| 5.00E-08 \| \| JS258995 \| 122 \| 85 \| Chr 14 \| 13432697 \| 13432781 \| 100 \| 6.00E-41 \| HIM-C14 \| 29 \| 100 \| 7.00E-13 \| \| HIM-C16 \| 29 \| 93 \| 4.00E-08 \| \| HIM-F157 \| 28 \| 92 \| 2.00E-07 \| \| JS267356 \| 123 \| 86 \| Chr 2 \| 7752027 \| 7752112 \| 100 \| 2.00E-41 \| HIM-C15 \| 21 \| 100 \| 5.00E-08 \| \| JS340458 \| 132 \| 99 \| Chr 18 \| 7868119 \| 7868217 \| 100 \| 3.00E-49 \| HIM-C14 \| 27 \| 100 \| 1.00E-11 \| \| HIM-C16 \| 30 \| 90 \| 3.00E-06 \| \| HIM-F157 \| 30 \| 96 \| 5.00E-11 \| \| JS399327 \| 139 \| 104 \| Chr 24 \| 11032953 \| 11033056 \| 100 \| 3.00E-52 \| HIM-C4 \| 26 \| 100 \| 5.00E-11 \| \| JS441932 \| 142 \| 107 \| Chr 16 \| 8527437 \| 8527543 \| 100 \| 5.00E-54 \| HIM-C10 \| 29 \| 96 \| 3.00E-10 \| \| JS558183 \| 150 \| 115 \| Chr 1 \| 812835 \| 812949 \| 100 \| 1.00E-58 \| HIM-C11 \| 30 \| 100 \| 2.00E-13 \| \| JS726283 \| 161 \| 129 \| Chr 17 \| 2306467 \| 2306595 \| 97 \| 7.00E-60 \| HIM-C13 \| 29 \| 100 \| 9.00E-13 \| \| JS774326 \| 174 \| 74 \| Chr 6 \| 2697040 \| 2697113 \| 98 \| 8.00E-32 \| HIM-F157 HIM-C16 HIM-C14 \| 29 29 29 \| 100 100 93 \| 1.00E-012 1.00E-012 6.00E-008 \| \| 74 \| Chr 7 \| 2355900 \| 2355973 \| 98 \| 8.00E-32 \| \| 74 \| Chr 9 \| 254485 \| 254558 \| 98 \| 8.00E-32 \| \| 74 \| Chr 10 \| 872221 \| 872294 \| 98 \| 8.00E-32 \| \| 74 \| Chr 12 \| 7120307 \| 7120380 \| 98 \| 8.00E-32 \| \| 74 \| Chr 16 \| 964612 \| 964685 \| 98 \| 8.00E-32 \| \| 13504369 \| 13504442 \| 98 \| 8.00E-32 \| \| 74 \| Chr 17 \| 4404909 \| 4404982 \| 98 \| 8.00E-32 \| \| 72 \| Chr 22 \| 12983318 \| 12983389 \| 98 \| 1.00E-30 \| \| 74 \| Chr 25 \| 4915130 \| 4915203 \| 98 \| 8.00E-32 \| \| 74 \| Chr 26 \| 4729308 \| 4729381 \| 97 \| 2.00E-29 \| \| 2024715 \| 2024788 \| 98 \| 8.00E-32 \| \| 74 \| Chr 29 \| 4619303 \| 4619376 \| 98 \| 8.00E-32 \| \| JS945511 \| 265 \| 123 \| Chr 14 \| 12914023 \| 12914145 \| 100 \| 3.00E-63 \| HIM-C16 HIM-F157 HIM-C14 \| 31 30 31 \| 100 100 93 \| 1.00E-013 4.00E-013 6.00E-009 \| \| 123 \| Chr 19 \| 6729355 \| 6729477 \| 100 \| 3.00E-63 \| \| 123 \| Chr 20 \| 7656105 \| 7656227 \| 100 \| 3.00E-63 \| \| 123 \| Chr 27 \| 14210419 \| 14210541 \| 100 \| 3.00E-63 \| \| JS1070115 \| 474 \| 440 \| Chr 9 \| 10758449 \| 10758777 \| 95.6 \| 1.00E-40 \| HIM-C14 \| 30 \| 100 \| 7.00E-13 \| \| HIM-C16 \| 30 \| 93 \| 4.00E-08 \| \| HIM-F157 \| 29 \| 93 \| 2.00E-07 \| \| JS511984 \| 146 \| 37 \| Chr 29 \| 14090259 \| 14090295 \| 100 \| 3.00E-12 \| HIM-C12 \| 29 \| 100 \| 2.00E-08 \| |
| --- | --- | --- | --- | --- | --- | --- | --- | --- | --- | --- | --- | --- | --- | --- | --- | --- | --- | --- | --- | --- | --- | --- | --- | --- | --- | --- | --- | --- | --- | --- | --- | --- | --- | --- | --- | --- | --- | --- | --- | --- | --- | --- | --- | --- | --- | --- | --- | --- | --- | --- | --- | --- | --- | --- | --- | --- | --- | --- | --- | --- | --- | --- | --- | --- | --- | --- | --- | --- | --- | --- | --- | --- | --- | --- | --- | --- | --- | --- | --- | --- | --- | --- | --- | --- | --- | --- | --- | --- | --- | --- | --- | --- | --- | --- | --- | --- | --- | --- | --- | --- | --- | --- | --- | --- | --- | --- | --- | --- | --- | --- | --- | --- | --- | --- | --- | --- | --- | --- | --- | --- | --- | --- | --- | --- | --- | --- | --- | --- | --- | --- | --- | --- | --- | --- | --- | --- | --- | --- | --- | --- | --- | --- | --- | --- | --- | --- | --- | --- | --- | --- | --- | --- | --- | --- | --- | --- | --- | --- | --- | --- | --- | --- | --- | --- | --- | --- | --- | --- | --- | --- | --- | --- | --- | --- | --- | --- | --- | --- | --- | --- | --- | --- | --- | --- | --- | --- | --- | --- | --- | --- | --- | --- | --- | --- | --- | --- | --- | --- | --- | --- | --- | --- | --- | --- | --- | --- | --- | --- | --- | --- | --- | --- | --- | --- | --- | --- | --- | --- | --- | --- | --- | --- | --- | --- | --- | --- | --- | --- | --- | --- | --- | --- | --- | --- | --- | --- | --- | --- | --- | --- | --- | --- | --- | --- | --- | --- | --- | --- | --- | --- | --- | --- | --- | --- | --- | --- | --- | --- | --- | --- | --- | --- | --- | --- | --- | --- |
