# Supplementary material for: Bracovirus Sneaks Into Apoptotic Bodies Transmitting Immunosuppressive Signaling Driven by Integration-Mediated eIF5A Hypusination
Source: Front Immunol. 2022 May 17;13:901593. doi: 10.3389/fimmu.2022.901593 (PMC9156803; doi:10.3389/fimmu.2022.901593)
Supplement: Supplementary Table 3 — Rich in proline glycine and charged amino acid proteins in Spodoptera litura hemocytes parasitized by Microplitis bicoloratus (Related to Figure 4 ). [file Table_3.docx]

| **Table S3 Rich in proline glycine and charged amino acid proteins in** Spodoptera litura hemocytes parasitised by Microplitis bicoloratus (Related to FIGURE 4) | | | | | | |
| --- | --- | --- | --- | --- | --- | --- |
| Sequenc ID | Protein Name | M/S result | Protein Mass | Protein Length | Special amino acid | % Cov (special amino acid） |
| XP_022822255.1 | CypA | Up | 22053.31 | 203 | 80 | 39.41 |
| XP_022825266.1 | CypD | Up | 17750.10 | 165 | 64 | 38.79 |
| XP_022835740.1 | CypJ | Up | 17892.19 | 161 | 64 | 39.75 |
| XP_022817682.1 | P53 | Up | 51410.20 | 472 | 215 | 45.55 |
| AOH69092.1 | Vank86 | Up | 19863.00 | 172 | 61 | 35.47 |
| YP_239406.1 | Vank92 | Up | 19167.88 | 167 | 61 | 36.53 |
| YP_239402.1 | Vank101 | Up | 21485.36 | 186 | 76 | 40.86 |
| XP_022828589.1 | eIF5A | / | 17524.84 | 160 | 71 | 44.38 |
| XP_022828595.1 | DHYS | Up | 41166.13 | 371 | 126 | 33.96 |
| XP_022830968.1 | DOHH | Up | 34099.50 | 304 | 116 | 38.16 |
